# The Augmented Cytopathologist: A Conceptual Exploratory Narrative Review on Immersive and Vision–Language Models Tools in Digital Pathology

**DOI:** 10.3390/jimaging12030100

**Published:** 2026-02-26

**Authors:** Enrico Giarnieri, Andrea Lastrucci, Alberto Ricci, Pierdonato Bruno, Daniele Giansanti

**Affiliations:** 1 Facoltà di Medicina e Psicologia, Sede Ospedale S. Andrea, Sapienza University of Rome, Via di Grottarossa 1035, 00189 Rome, Italy; enrico.giarnieri@uniroma1.it; 2 Dipartimento Professioni Tecnico Sanitarie e della Riabilitazione, Azienda Ospedaliero-Universitaria Careggi, 50134 Florence, Italy; andrea.lastrucci@unifi.it; 3 Respiratory Disease Unit, Sant’Andrea University Hospital, Sapienza University of Rome, Via di Grottarossa 1035, 00189 Rome, Italy; alberto.ricci@uniroma1.it (A.R.); pierdo.bruno@gmail.com (P.B.); 4 Centro Nazionale IAHTA, Istituto Superiore di Sanità, Via Regina Elena 299, 00161 Rome, Italy

**Keywords:** cytopathology, augmented reality, virtual reality, LLM, VLM, digital pathology, large language model, digital cytology, cytology

## Abstract

Emerging digital technologies, including immersive environments (VR/AR/XR) and Vision–Language Models (VLMs), have the potential to reshape digital pathology and medical imaging. While immersive tools can enhance spatial visualization and procedural training, VLM-based copilots offer cognitive and workflow support. Their combined impact on cytopathology remains largely conceptual and preclinical. This Conceptual Exploratory Narrative Review (CENR) examines how immersive technologies and VLM-based copilots may jointly influence cytopathologists’ professional workflow, training, and diagnostic processes, introducing the notion of the “augmented cytopathologist.” A structured exploratory approach integrated peer-reviewed literature, position papers, preprints, gray literature (technical reports, white papers, conference abstracts, blogs), and cross-disciplinary perspectives. Database searches (PubMed, Web of Science, Scopus) confirmed a limited number of studies addressing immersive or AI-assisted cytopathology imaging. Thematic analysis focused on four conceptual dimensions: (1) technological capabilities and maturity; (2) workflow and educational applications; (3) professional implications and cytopathologist role; and (4) responsible use of LLMs and VLMs as supportive tools. This approach emphasizes interpretation of emerging trends over aggregation of empirical data, enabling conceptual synthesis of early-stage implementations and perspectives in the field. Immersive technologies facilitate three-dimensional visualization, procedural skill development, and collaborative engagement, whereas VLMs support report generation, literature retrieval, and decision guidance. Together, they offer a synergistic model for perceptual and cognitive augmentation. Key challenges include technical maturity, interoperability, workflow integration, regulatory compliance, and ethical oversight. Figures illustrate representative examples of (1) remote collaborative immersive evaluation and (2) integration of immersive visualization with VLM-based copilots, highlighting potential applications in training and workflow support. The CENR underscores the potential of combining immersive tools and AI copilots to support cytopathology, particularly for education, workflow efficiency, and cognitive augmentation. Adoption should be incremental and carefully governed, emphasizing augmentative rather than transformative use. Future research should focus on clinical validation, scalable integration, and regulatory and ethical frameworks to realize the concept of the augmented cytopathologist in practice.

## 1. Introduction

### 1.1. From Conventional Cytopathology to Digital and AI-Driven Transformation

Cytopathology has always been a discipline grounded in visual expertise and interpretive judgment. Since the mid-20th century, it has evolved from conventional smears and manual microscopy toward automated staining, liquid-based cytology, and digital archiving. Despite these technological advances, the core of cytopathological practice remains deeply human: the cytopathologist continuously integrates visual patterns with clinical context, often under conditions of uncertainty and limited tissue availability.

The digital transformation of cytopathology has unfolded progressively over the past two decades, building on early telepathology experiences developed in the late 20th century [1]. Initial efforts focused on the transmission of static images [2,3], with Petrovichev et al. exploring their diagnostic applicability in cytology [3]. Subsequent advances introduced dynamic telepathology systems with robotic control, enabling real-time remote slide examination, as demonstrated by Della Mea et al. [4]. Integrated networks combining robotic and non-robotic telepathology modalities were later described by Dunn et al. [5], while Demichelis et al. confirmed the feasibility of robotic telepathology for intraoperative consultations using still images [6]. Nevertheless, communication latency remained a significant limitation in these systems, as reported by Carr et al. [7].

Virtual microscopy represented a key step in overcoming these constraints. The transition from physical slides to high-resolution digital replicas accessible remotely was documented by Giansanti et al. [1]. Educational platforms such as virtual-microscopy.net [8], histologyguide.com [9], and virtualmicroscopy.co.uk [10] further supported this transition by providing structured repositories and training resources. Donnelly et al. demonstrated the successful adoption of virtual microscopy in cytotechnology education [11], while Saini et al. reviewed the expanding role of Whole Slide Imaging (WSI) in digital cytology [12]. Additional educational and training benefits were highlighted by Chiou and Jia [13], and by Giansanti et al., who described the integration of virtual microscopy into telepathology training programs to strengthen digital competencies [14].

Despite these advances [14,15], digital cytopathology presents challenges that are distinct from those encountered in histopathology. Cytological specimens are inherently heterogeneous and three-dimensional, often requiring multilayer imaging techniques such as Z-stacking to accurately capture cellular depth [16]. Software-based focus enhancement has been proposed to improve image clarity in digital cytology [17], yet implementation of digital workflows frequently entails increased initial workload and infrastructure costs. Survey data from Denmark reported by Smith et al. indicate that the transition to digital pathology can place significant strain on organizational processes [15], a finding echoed by Detlefsen et al. [18]. At the same time, the growing integration of artificial intelligence has added further complexity to digital pathology workflows [19]. Standardization remains a critical issue, as emphasized by Marletta et al. through a Delphi consensus focused on WSI in thyroid cytopathology [20], while Chantziantoniou et al. identified barriers such as pathologist resistance and algorithmic limitations that continue to slow adoption [21]. Regulatory frameworks, including FDA guidelines on WSI, play a central role in ensuring quality, consistency, and compliance during this transition [22].

Within this evolving digital landscape, artificial intelligence has emerged as a major driver of transformation in cytopathology. AI-based systems have demonstrated their ability to enhance diagnostic accuracy and workflow efficiency by supporting cellular image analysis and algorithmic classification [23,24]. Beyond image interpretation, AI has expanded into predictive analytics, enabling disease progression forecasting and supporting clinical decision-making [25]. In high-volume screening settings, AI-driven systems have contributed to a substantial reduction in manual workload for cytotechnologists while maintaining high diagnostic standards [26]. However, the heterogeneity of cytological samples, characterized by variable staining and complex three-dimensional structures, complicates algorithm training and limits model generalizability [27]. Effective deployment therefore requires robust and diverse datasets, as well as architectures capable of adapting to real-world variability [28].

Equally important is the integration of AI into routine clinical workflows. AI tools must align with established diagnostic practices and support, rather than disrupt, the work of cytopathologists. Achieving this alignment requires iterative validation and close collaboration between clinicians, computer scientists, and system developers [29]. While AI holds considerable promise, it has not yet reached full maturity in cytopathology. As highlighted in comprehensive reviews by Giansanti [30,31], the acceleration of digital cytopathology—particularly during the COVID-19 pandemic—has laid the technological and cultural foundations for AI-driven innovation, but ethical, regulatory, and organizational challenges remain. Digitization and AI should therefore be understood as deeply interconnected processes within a dynamic and still-evolving transformation, rather than as sequential or fully stabilized paradigms.

### 1.2. Immersive Technologies and Vision–Language Models as Emerging Copilots

Building on the combined trajectories of digitization and artificial intelligence, a further layer of innovation is emerging through the integration of immersive technologies and Vision–Language Models (VLMs), including large language models (LLMs) [32,33]. Immersive technologies—such as augmented reality (AR), virtual reality (VR), extended reality (XR), and metaverse-based platforms—function as advanced human–machine interfaces rather than AI per se. Their primary value lies in enhancing spatial understanding, interactive training, real-time collaboration, and contextual data immersion, particularly in complex imaging and educational scenarios relevant to cytopathology.

The development of immersive technologies reflects the progressive evolution of both hardware and software ecosystems. Advances in head-mounted displays, controllers, hand-tracking systems, haptic interfaces, and rendering algorithms have addressed earlier limitations related to computational power, display resolution, and user discomfort. Among current platforms, the HoloLens 2 (Microsoft) represents one of the most advanced AR/MR devices in healthcare, integrating holographic overlays, spatial audio, tracking sensors, and wireless connectivity. Its applications in surgical navigation, anatomical education, and medical training have been highlighted by Palumbo [34], while Veerla et al. demonstrated the potential of combining mixed reality visualization with VLM integration to optimize digital pathology workflows and collaborative diagnostics [35].

Other immersive platforms are increasingly explored in digital pathology and medical education. The Oculus Rift has been explored for the examination of digital pathology slides [33], while the Apple Vision Pro (Apple) has been proposed as a paradigm shift in spatial computing for medical visualization and training [36]. More accessible solutions such as the Valve Index (Valve), Google Cardboard (Google), and Samsung GearVR (Samsung) are commonly adopted in educational and preliminary simulation settings, although further validation is needed for sustained clinical use. Beyond education, immersive technologies have demonstrated broader clinical utility, including enhanced teaching of three-dimensional anatomy and pathology [37] and applications in pain management during medical procedures [38].

Table 1 summarizes key immersive devices, highlighting their primary technological features, typical applications in healthcare and education, and the expected benefits for user training, workflow optimization, and spatial comprehension. Rather than presenting an exhaustive list, the table emphasizes illustrative platforms that are representative of current trends in AR/VR adoption for biomedical purposes, allowing readers to grasp the diversity of hardware capabilities and their relevance to cytopathology and related clinical contexts.

In parallel, VLM-based copilots are rapidly transforming biomedical research and healthcare by providing advanced language understanding, reasoning capabilities, and context-aware assistance. Leveraging natural language processing, these systems can synthesize large bodies of biomedical literature, integrate multimodal data, and generate actionable insights that support clinical and research decision-making. Platforms such as Med-PaLM 2 (Google DeepMind), Claude (Anthropic), ChatGPT 5.2 (OpenAI), and BioGPT (Microsoft Research) illustrate how VLMs can support literature summarization, guideline interpretation, hypothesis generation, and decision support across biomedical domains [45,46,47,48].

Table 2 presents representative VLM platforms with their main functionalities, application domains, and demonstrated benefits. The table is designed to provide a conceptual overview of how these AI copilots can assist healthcare professionals and researchers in complex decision-making tasks, facilitating literature synthesis, evidence-based recommendations, and integration with digital workflows. Again, the examples are illustrative rather than exhaustive, underscoring the ongoing development and necessary human oversight associated with these technologies.

When integrated with digital infrastructures such as electronic health records, laboratory information systems, and AI-powered imaging platforms, VLM copilots have the potential to form cohesive ecosystems that bridge the gap between raw data and actionable knowledge. However, these systems remain illustrative examples rather than exhaustive solutions, and their deployment continues to require careful human oversight to ensure accuracy, transparency, and clinical safety.

While VLMs, including LLM-enabled systems, hold significant promise in supporting literature synthesis, evidence integration, and workflow facilitation, it is crucial to recognize that these models are not designed for autonomous medical diagnosis. Their use should remain augmentative, providing clinicians and cytopathologists with decision support while maintaining human oversight, adherence to established clinical standards, and reliance on domain-specific medical models. Techniques such as retrieval-augmented generation (RAG) can offer context-sensitive information, yet they cannot replace expert judgment or fully validated diagnostic systems.

At the same time, the broader market trajectory reflects rapid and largely unstoppable growth. The global immersive technology market—including AR, VR, and MR—is projected to increase from approximately USD 1.67 billion in 2025 to over USD 9 billion by 2035, representing a compound annual growth rate (CAGR) of ~18.5% [49]. Similarly, the healthcare AI market, covering machine learning, natural language processing, and LLM-enabled systems, is expected to expand at a CAGR of ~35% over the next decade [50], driven by increasing adoption in diagnostics, decision support, and clinical workflows. This dual perspective highlights that while clinical caution remains essential, the technological and economic momentum of immersive and AI-enabled systems is accelerating decisively, shaping the future landscape of cytopathology and biomedical practice.

### 1.3. Objective and Structure of the Conceptual Exploratory Narrative Review

In this emerging fields several questions naturally arise: How are immersive technologies being applied in healthcare and cytopathology, and what benefits do they provide for training, diagnostics, and workflow optimization? In what ways can VLM-based copilots support clinical decision-making, report generation, and cognitive augmentation in cytopathology? What technical, operational, and workflow challenges must be addressed to integrate these tools into routine practice? How do ethical, regulatory, and organizational factors influence their safe and effective adoption? And, finally, how is the role of the cytopathologist evolving in response to immersive tools and AI copilots, and what implications emerge for professional competencies and training?

In this conceptual exploratory narrative review (CENR), we address these questions by examining how immersive technologies and VLM-based AI copilots may jointly transform cytopathological practice and training. The CENR approach integrates insights from peer-reviewed literature, preprints, gray sources, and digital platforms to propose an interpretive framework in domains where empirical evidence is still limited. Traditional systematic or scoping reviews are often impractical in emerging areas due to the scarcity of consolidated studies. A conceptual exploratory approach enables the identification of trends, challenges, and opportunities, while supporting interdisciplinary synthesis and forward-looking interpretation.

We focus on two complementary technological strands: immersive technologies, which enhance spatial understanding and procedural skills through interactive three-dimensional visualization, and VLMs, which provide cognitive augmentation by supporting interpretation, report generation, and knowledge synthesis. Their convergence underpins the concept of the “augmented cytopathologist,” a professional who integrates morphological expertise with digital literacy and AI-assisted decision support.

The remainder of the manuscript is organized as follows. Section 2 describes the study design and conceptual approach. Section 3 presents current applications of immersive technologies and VLM copilots in healthcare and cytopathology. Section 4 critically discusses technical, operational, ethical, and professional implications. Section 5 presents an integrative conceptual framework synthesizing insights from immersive technologies and AI copilots in cytopathology. Section 6 discusses the limitation and the future work. Section 7 is dedicated to the conclusions.

## 2. Methodological Approach

### 2.1. Study Design and Conceptual Approach

This CENR adopts an exploratory and integrative approach to describe the emerging role of immersive technologies and Vision–Language Models (VLMs), including large language models (LLMs), as copilots in cytopathology. Rather than generating new empirical findings, the goal is to provide an interpretation of ongoing developments, integrating evidence from multiple sources and disciplinary perspectives. This approach is typical of CENRs, which are particularly suitable for areas where empirical literature is sparse and early-stage implementations are limited, allowing conceptual synthesis and framework development.

The CENR follows the editorial guidelines of the journal [51], which specify that “The structure can include an Abstract, Keywords, Introduction, Relevant Sections, Discussion, Conclusions, and Future Directions.” No specific protocol or detailed methodology is required, yet in this section we provide a methodological orientation designed to reveal emerging themes, critical challenges, and conceptual patterns in the integration of immersive technologies and AI copilots into cytopathology, rather than to generate exhaustive empirical data.

Traditional bibliographic databases such as PubMed, Web of Science, and Scopus yielded very limited results, reflecting the early stage of research on immersive simulations and AI copilots in cytopathology. Representative PubMed searches conducted in October 2025 illustrate this scarcity:

- LLMs and copilots in cytopathology:
  Query: ((LLM[Title/Abstract]) AND (copilot[Title/Abstract])) AND (cytopathology[Title/Abstract]).
  PubMed link → L1 → 0 results.
- Immersive tools in cytopathology:
  Query: ((Virtual reality[Title/Abstract]) OR (metaverse[Title/Abstract]) OR (Augmented Reality[Title/Abstract])) AND (cytopathology[Title/Abstract]).
  PubMed link → L2 → 1 result.

These results clearly indicate that traditional systematic review methods would have been inadequate, as they rely on substantial published empirical evidence. In contrast, a CENR allows for a more flexible and integrative synthesis, capturing early-stage developments, conceptual frameworks, and pilot implementations that are not yet peer-reviewed or formally reported.

To complement these findings, the review also included preprints (arXiv, medRxiv), gray literature (technical reports, white papers, conference abstracts, blogs), and cross-disciplinary sources (computer science, medical informatics, digital pathology, education, human–technology interaction). This combination allowed the capture of early experimental implementations, pilot projects, and emerging discussions in the field, providing a more comprehensive understanding of the state of the art. The investigation draws on three main domains:

- Scientific and Technical Literature: Publications from 2020 onward addressing immersive technologies (VR, AR, XR, metaverse) and VLM-based copilots in healthcare, with emphasis on cytopathology and digital pathology, were included. Boolean-based searches enabled focused exploration of both technological innovation and workflow integration.
- Gray Literature and Digital Platforms: Technical reports, white papers, conference abstracts, blogs, and curated online repositories were included to document early implementations and pilot projects in education and diagnostic practice.
- Cross-Disciplinary Perspectives: Insights from computer science, medical informatics, digital pathology, education, and human–technology interaction were integrated to highlight potential benefits, tensions, and implications for professional roles, training models, and human–AI collaboration.

Overall, the CENR approach is justified by the scarcity of formal evidence, the early stage of technological development, and the cross-disciplinary nature of the field. By integrating preprints, gray literature, and conceptual discussions, this review provides a foundational synthesis that can guide future empirical research, workflow design, and educational strategies in cytopathology.

Table 3 summarizes the key domains, search keywords, and the scope of inquiry, illustrating how each source contributes to understanding emerging immersive and AI-assisted tools in cytopathology workflows. Table 4 expands on the range of sources beyond traditional peer-reviewed literature, including preprints, gray literature, cross-disciplinary perspectives, and software/package repositories. This broader view highlights the diverse origins of emerging evidence, capturing pilot implementations, experimental platforms, and early-stage discussions that are critical to an integrative understanding of immersive technologies and AI copilots in cytopathology.

The analysis followed a thematic and iterative approach to explore how immersive technologies and VLM-based copilots are represented in healthcare and cytopathology. Rather than aggregating empirical results, the focus was on identifying key conceptual dimensions relevant to the research questions:

1. Technological Capabilities and Maturity
  Examining hardware, software, and platform developments to understand readiness for integration and potential benefits for training, diagnostics, and workflow optimization.
2. Workflow and Educational Applications
  Exploring how these tools may support clinical and educational processes, including cytopathology training, remote collaboration, and report generation, while noting practical and operational constraints.
3. Professional Implications and Cytopathologist Role
  Considering how AI and immersive technologies may influence expertise, autonomy, and professional competencies, and how the cytopathologist’s role may evolve in response to digital augmentation.
4. Responsible Use of LLMs and Copilots
  Framing LLMs and copilots as supportive, augmentative tools rather than autonomous diagnostic systems, focusing on conceptual understanding of their role, applications, and boundaries.

This approach allowed for a broad interpretive synthesis, providing a framework to discuss the applications, benefits, challenges, and professional implications of immersive technologies and AI copilots in cytopathology, aligned with the review’s guiding questions.

### 2.2. Study Selection Flow

The selection of sources from those listed in Table 4 followed a concept-driven, iterative approach consistent with the CENR methodology. Rather than applying strict criteria of methodological quality, the focus was on relevance to key conceptual domains: technological innovation, workflow integration, educational applications, and professional implications.

D.G. and E.G. independently screened all candidate sources, including peer-reviewed papers, preprints, gray literature, cross-disciplinary studies, and software/package repositories. Each source was evaluated for its ability to inform the conceptual framework of immersive technologies and AI copilots in cytopathology. For every source ultimately selected, a brief consensus report (CR) was drafted to document the rationale for inclusion and ensure traceability of decisions. Any disagreements between reviewers were resolved through discussion and consensus.

The selection flow can be summarized as follows:

1. Identification: Retrieval of all candidate sources from bibliographic databases, preprint servers, gray literature, and cross-disciplinary platforms.
2. Relevance Screening: Assessment of titles, abstracts, or executive summaries for alignment with the conceptual domains:
  ○ Technological capabilities and maturity;
  ○ Workflow and educational applications;
  ○ Professional implications and cytopathologist role;
  ○ AI copilots and immersive systems in cytopathology.
3. Inclusion in Analysis: Sources contributing meaningful insights to at least one domain were retained for thematic synthesis.

This approach ensured that the CENR captured the diversity of emerging discussions, pilot implementations, and experimental platforms, while remaining faithful to its exploratory and integrative objectives. The use of consensus reports for each selected study further strengthens transparency and rigor in documenting how conceptually relevant sources were identified and retained. 

Additional details on the study selection process are reported in the Supplementary Materials (see Supplementary Materials section). In total, 26 references [52,53,54,55,56,57,58,59,60,61,62,63,64,65,66,67,68,69,70,71,72,73,74,75,76,77] were selected and included in the results section.

## 3. Results

### 3.1. Exploring Immersive Healthcare Solutions: A Pathway to Digital Cytopathology Integrations

Immersive technologies, encompassing augmented reality (AR), virtual reality (VR), and mixed reality (MR), are transforming healthcare education, procedural training, and diagnostic workflows. Their core strength lies in creating intuitive, interactive environments in which anatomical structures, instruments, and procedural steps can be visualized in three dimensions. These platforms, accessible via VR headsets, AR-enabled devices, or even smartphones, allow learners to acquire foundational and advanced skills in a controlled setting before engaging with real patients. By enabling rehearsal of complex preoperative procedures, including detailed anatomical navigation and sequential operational steps, immersive systems enhance both cognitive and procedural understanding [52]. Compared to traditional textbooks, 2D images, or procedural videos, immersive environments provide richer sensory engagement, combining visual, spatial, and haptic cues, which support long-term knowledge retention and deeper comprehension of complex tasks.

Beyond individual skill acquisition, immersive simulations foster interdisciplinary learning and collaboration among healthcare teams. Integration with AI-driven modules enables real-time feedback, adaptive difficulty adjustment, and performance tracking, reinforcing patient safety and procedural accuracy [53]. Haptic-enabled VR systems further enhance this learning by simulating tactile interactions with tissues and instruments, promoting manual dexterity and coordination within risk-free scenarios [54]. In parallel, patient-centered applications of immersive technologies offer interactive educational experiences that reduce anxiety, improve understanding of procedures, and enhance compliance, particularly in pediatric and geriatric populations [55].

Building on these foundations, immersive technologies are progressively applied to cytopathology education and diagnostic workflows. Virtual microscopy and whole-slide imaging (WSI) platforms allow high-resolution scanning and remote examination of cytology slides, enabling telecytology and distance learning. Commercial solutions such as Leica Aperio, Philips IntelliSite, and Hamamatsu scanners provide high-quality digitized slides, supporting accurate diagnosis and structured educational interactions [56]. Optimized slide preparation and scanning improve image fidelity, which is critical for both diagnostic accuracy and effective training outcomes [56].

Beyond slide visualization, fully simulated laboratory environments are being developed. Immersive replicas of cytopathology labs allow trainees to practice fine-needle aspirations (FNAs), slide preparation, staining techniques, artifact recognition, and rapid on-site evaluations before performing real procedures [57,58,59]. Such simulations enhance procedural consistency, reproducibility, and confidence while minimizing risk and accelerating learning curves. Advanced AR/VR systems introduce interactive 3D cellular models, allowing exploration of spatial and morphological relationships that are difficult to perceive with conventional slides alone [59]. Integrative platforms like Microscope 2.0 overlay AI-generated heatmaps and annotations directly into the pathologist’s field of view, combining immersive visualization with real-time interpretive support [60]. These tools create a continuous learning environment where novices and experienced cytopathologists alike can refine their skills and optimize workflow efficiency.

Taken together, immersive healthcare solutions establish a layered pathway to digital cytopathology. They begin with foundational skill acquisition through interactive simulation, progress to collaborative and interdisciplinary learning, and culminate in application within digital laboratory environments where AI and AR/VR technologies converge. By bridging educational, procedural, and diagnostic domains, these platforms enhance safety, efficiency, and accuracy, offering a cohesive framework for integrating emerging technologies into cytopathology practice.

#### 3.1.1. General Applications of Immersive Technologies in Healthcare

Immersive technologies—including augmented reality (AR), virtual reality (VR), and mixed reality (MR)—are increasingly used in healthcare to provide interactive, intuitive, and flexible learning environments. By enabling visualization of anatomical structures, instruments, and procedural steps in three dimensions, these platforms offer learners the opportunity to acquire both foundational and advanced clinical skills before interacting with real patients, minimizing risk and reducing cognitive load [52]. These immersive experiences allow progressive skill development, enabling trainees to rehearse complex procedures, explore surgical access routes, and internalize sequential steps in a controlled, repeatable setting.

Unlike traditional learning resources such as textbooks, 2D images, or instructional videos, immersive systems combine visual, spatial, and sometimes haptic cues, providing a multisensory experience that enhances engagement, comprehension, and long-term retention. For instance, haptic-enabled VR platforms simulate tactile interactions with tissues or surgical instruments, allowing learners to refine manual dexterity, hand–eye coordination, and procedural fluency in a risk-free environment [54]. This form of simulation is particularly valuable for procedures that require precise manipulations or fine motor skills, which are difficult to practice safely in clinical settings.

Immersive technologies also facilitate interdisciplinary learning, promoting teamwork, communication, and coordination among different healthcare professionals. When integrated with AI-driven modules, immersive platforms can provide real-time feedback, adaptive difficulty adjustment, and performance tracking, reinforcing best practices and patient safety principles [53]. By simulating realistic clinical scenarios, these tools help learners anticipate potential challenges, troubleshoot errors, and internalize standardized protocols, fostering a culture of safety and collaborative problem-solving.

Beyond training healthcare professionals, immersive technologies are increasingly applied for patient education. Interactive AR/VR experiences can help patients, particularly pediatric or geriatric populations, understand the rationale, steps, and expected outcomes of medical procedures. By providing clear visualizations of anatomy, instrumentation, and procedural sequences, these tools can reduce anxiety, improve comprehension, and increase cooperation during interventions [55]. Such patient-centered approaches support shared decision-making and enhance the overall healthcare experience.

To conceptualize the collective impact of immersive technologies, we introduce the Augmented Workforce Training Group (AWTG) framework. The AWTG highlights four key domains where immersive systems contribute to healthcare:

1. Skill acquisition and procedural training: enabling interactive rehearsal, repetitive practice, and progressive mastery of clinical tasks [52];
2. Interdisciplinary collaboration: fostering teamwork, communication, and adherence to patient safety principles [53];
3. Realistic simulation: providing tactile, interactive, and haptic-enabled practice for surgical and procedural training [54];
4. Patient engagement and education: enhancing understanding, reducing anxiety, and improving compliance through immersive educational experiences [55].

While these immersive technologies show clear promise in enhancing training, skill retention, and patient experience, it is important to approach them with cautious optimism. Most current implementations serve as supplementary educational and workflow tools rather than replacements for clinical judgment or hands-on experience. Effectiveness depends on the quality of content, realism of simulation, and integration into broader clinical education programs, emphasizing that immersive platforms are enablers rather than autonomous solutions.

Overall, the integration of immersive technologies establishes a structured, safe, and interactive learning pathway that bridges theoretical knowledge, technical skills, and patient-centered practice, providing the conceptual foundation for more specialized applications, such as in digital cytopathology.

#### 3.1.2. Immersive Technologies in Digital Cytopathology: From Visualization to Procedural Mastery

Immersive technologies, including augmented reality (AR), virtual reality (VR), and mixed reality (MR), are progressively transforming cytopathology education and laboratory workflows by providing interactive, highly visual, and context-rich environments. These systems enable trainees and professionals to explore cellular morphology, tissue architecture, and laboratory procedures in three dimensions, significantly enhancing spatial understanding, procedural comprehension, and interpretive reasoning beyond conventional 2D images or video-based learning [56,57,58,59,60].

A principal advantage of these immersive platforms is their ability to simulate complete laboratory environments, from slide preparation and staining protocols to fine-needle aspirations (FNAs) and on-site evaluations. By digitally replicating both routine and complex tasks, immersive systems create a safe, repeatable, and risk-free environment where learners can develop technical dexterity, troubleshoot errors, and internalize standard operating procedures before engaging with real specimens [57,58]. Studies indicate that such simulations improve procedural consistency, confidence, and efficiency, fostering accuracy and reliability in laboratory practice [59].

Beyond procedural rehearsal, immersive technologies provide enhanced interaction with cellular and tissue structures. Advanced AR/VR tools allow the manipulation of 3D cellular models, revealing spatial and morphological relationships that are challenging to perceive using traditional microscopy alone [59]. Some platforms integrate real-time visual overlays, guiding users through complex analyses and highlighting key diagnostic features for educational purposes [60]. These interactive experiences support the development of critical observation skills, pattern recognition, and interpretive reasoning, reinforcing structured learning while maintaining controlled, low-risk conditions.

Immersive platforms also facilitate team-based and interdisciplinary learning, promoting collaboration among cytotechnologists, pathologists, and other laboratory personnel. By simulating integrated laboratory workflows, shared case discussions, and coordinated diagnostic decision-making, these technologies enhance communication, workflow understanding, and adherence to procedural standards, contributing to both educational and operational improvements [53,56].

Additionally, immersive technologies can be applied to patient engagement and education, particularly for procedures that are complex or anxiety-inducing. Interactive 3D visualizations allow patients to understand procedural steps, laboratory workflows, and expected outcomes, improving comprehension, reducing fear, and enhancing compliance with clinical protocols [55]. While the primary focus here remains on cytopathology, these examples illustrate a broader principle: immersive platforms offer multi-sensory, interactive, and context-aware experiences that bridge the gap between theoretical knowledge, technical skill, and practical application [52,53,54,55].

It is, however, important to make the appropriate considerations regarding boundaries.

Despite their advantages, immersive technologies have well-defined limits that must be acknowledged. First, these systems do not replace hands-on experience with actual specimens, nor can they fully substitute the nuanced judgment of experienced cytopathologists. Their strength lies in preparatory training, procedural rehearsal, and enhanced visualization, but diagnostic authority remains with human experts.

Second, immersive platforms are dependent on data quality and fidelity. High-resolution imaging, accurate 3D reconstruction, and realistic simulation of tactile interactions (in haptic-enabled systems) are crucial; poor input quality can propagate errors in learning or misrepresent anatomical and cellular structures [56,57].

Third, technological and logistical constraints, including hardware cost, software compatibility, and infrastructure requirements, may limit widespread adoption, particularly in resource-constrained settings. Careful integration into curricula and workflows is necessary to ensure practical feasibility and sustainability.

Finally, immersive systems should be considered complementary to, not substitutive of, AI-assisted diagnostic tools or traditional teaching methods. While AR/VR can enhance perception, spatial understanding, and procedural rehearsal, they do not inherently perform analysis, generate diagnoses, or replace professional judgment. Future integration with AI-assisted guidance, workflow optimization, or tool-augmented pipelines should be approached cautiously, maintaining human oversight and educational primacy [56,57,58,59,60].

In sum, immersive technologies in digital cytopathology act as comprehensive educational and procedural scaffolds, supporting visualization, procedural rehearsal, interdisciplinary collaboration, and patient engagement. By acknowledging their boundaries, these platforms provide a safe, progressive, and context-aware learning pathway, laying the foundation for future, carefully layered integrations with AI and other digital tools while preserving accuracy, reproducibility, and human oversight [56,57,58,59,60].

Table 5 provides a structured synthesis of key studies that have contributed to understanding the role of immersive and digitally mediated technologies in cytopathology training, laboratory workflows, and patient engagement. While some studies focus on clearly immersive modalities such as virtual reality (VR), augmented reality (AR), and haptic simulations, others [56,57,58] explore virtual digital reconstruction, slide scanning, and computational platforms that, although not immersive per se, provide essential high-fidelity digital environments supporting spatial understanding, procedural rehearsal, and workflow optimization.

This collection of studies highlights both the opportunities and the boundaries of immersive and digital technologies. Immersive tools enhance procedural training, visualization of cellular and tissue structures, and interdisciplinary collaboration, yet they do not replace hands-on experience or the judgment of experienced cytopathologists. Digital and computational tools further contribute by enabling accurate data capture, repeatable workflows, and safe practice, forming the broader ecosystem in which immersive experiences can be effectively integrated.

By combining these insights, Table 5 summarizes the technologies, contexts, purposes, and observed boundaries of the reviewed studies, providing a clear overview of the landscape that underpins the subsequent discussion on immersive technologies in digital cytopathology.

### 3.2. Integrating Domain-Specific LLMs and VLMs for Workflow Support in Digital Pathology

#### 3.2.1. Limits of LLMs and VLMs in Direct Medical Diagnosis

The reviewed literature consistently emphasizes that large language models (LLMs), including vision–language models (VLMs), are not designed to function as autonomous diagnostic systems in clinical medicine, digital pathology, or cytopathology. Across multiple clinical domains, LLM-based systems have demonstrated utility primarily as decision-support and reasoning aids rather than as diagnostic authorities. Reviews and perspective articles explicitly frame LLMs as tools for enhancing, rather than replacing, human expertise, highlighting their role in synthesizing guidelines, structuring clinical information, and supporting complex decision-making processes [61].

Empirical studies evaluating LLMs in diagnostic contexts report encouraging performance in structured or simulated settings, but also delineate clear boundaries of applicability. GPT-4–based systems have shown the ability to generate differential diagnoses, summarize longitudinal patient records, and assist in complex case reasoning when provided with curated clinical information [63]. Similarly, guideline-aware implementations, such as those aligned with National Comprehensive Cancer Network (NCCN) protocols, demonstrate value in reinforcing adherence to standardized care pathways, particularly in oncology decision support [62]. However, these systems operate by reasoning over provided abstractions—clinical text, structured inputs, or summarized findings—rather than by extracting diagnostic features directly from raw medical data.

This distinction is further reinforced by evaluations of LLM performance on professional examination-style tasks. Comparative analyses of LLMs on specialty board exam questions in the health domain reveal strong performance in knowledge recall and protocol-based reasoning, yet also expose vulnerabilities to hallucination, contextual misinterpretation, and lack of causal grounding, underscoring their unsuitability for unsupervised clinical diagnosis [63]. Together, these findings indicate that LLM competence is tightly coupled to the structure, completeness, and framing of the input information, rather than to independent diagnostic inference.

The limitations of LLMs become particularly evident in pathology and cytopathology, where diagnosis depends on fine-grained morphological patterns, spatial relationships among cells, and tissue architecture that are not readily reducible to textual descriptors. Even in multimodal settings, general-purpose VLMs rely on visual encoders that are typically less specialized than dedicated pathology models. As shown in studies of multimodal pathology assistants, diagnostic reliability arises from large-scale, domain-specific visual foundation models, while the language component primarily supports interpretation, explanation, and reporting [64,65].

PathChat and TITAN exemplify this paradigm, demonstrating that clinically meaningful performance in digital pathology is achieved through pretrained whole-slide visual encoders, with LLMs serving as interactive and explanatory layers rather than as primary diagnostic engines [65]. Similarly, analyses of generalist multimodal chatbot systems deployed as pathology copilots reveal systematic pitfalls, including reduced robustness to rare entities and sensitivity to prompt formulation, further cautioning against their use as standalone diagnostic tools [67].

In cytopathology, where datasets are smaller and morphological variability is high, these limitations are even more pronounced. Architectures such as GNNFormer explicitly model cellular morphology and spatial organization using graph-based representations, illustrating that accurate cytopathological reasoning requires inductive biases and model structures tailored to biological organization, well beyond the capabilities of general-purpose LLMs or loosely coupled VLMs [66].

From a clinical governance and regulatory standpoint, these findings converge on a consistent conclusion: expecting LLMs or VLMs to perform standalone medical diagnosis is both conceptually and practically inappropriate. Their probabilistic, generative nature, combined with sensitivity to input framing and lack of intrinsic grounding in biological signal extraction, conflicts with requirements for reproducibility, traceability, and clinical validation. Accordingly, their appropriate role lies in collaborative, tool-augmented pipelines, where diagnostic-grade neural networks trained on domain-specific data perform core inference, and LLMs provide orchestration, contextual reasoning, and communication support [61,62,63,64,65,66,67]. Smaller neural networks specialized for specific cytological or pathological tasks can be integrated as modular tools, enhancing the pipeline’s versatility and diagnostic reliability.

#### 3.2.2. Foundation Models and Tool-Augmented Architectures in Digital Pathology

Recent advances in computational pathology have been driven primarily by the emergence of large-scale, domain-specific foundation models trained on whole-slide images (WSIs) and paired visual–textual data. Unlike general-purpose or pathology-adapted VLMs, these foundation models are explicitly designed to capture the multi-scale morphological, spatial, and contextual features that underpin diagnostic reasoning in histopathology and cytopathology. By learning pathology-native visual representations directly from large and heterogeneous datasets, they provide a robust substrate for downstream diagnostic, prognostic, and reporting tasks, reducing reliance on narrowly optimized, task-specific architectures.

This shift marks a transition from task-centric modeling to representation-centric learning in computational pathology. Foundation models emphasize generalizable visual embeddings that can be reused across multiple clinical applications, while language components are introduced selectively to enhance interpretability, interaction, and workflow integration rather than to perform primary diagnostic inference. General-purpose LLMs, such as ChatGPT, serve here mainly as benchmarks to illustrate reasoning capabilities and workflow integration; they are not medically reliable by design.

##### Domain-Specific Foundation Models

CONCH represents a paradigmatic example of a pathology-native visual–language foundation model. It leverages contrastive learning over more than 1.17 million image–caption pairs derived from histopathology images and biomedical text, enabling task-agnostic pretraining that yields highly transferable representations [68]. CONCH demonstrates strong performance across a wide range of downstream tasks—including classification, segmentation, captioning, and cross-modal retrieval—illustrating how diagnostic versatility emerges from robust visual representations rather than from task-specific optimization.

PathOrchestra further extends this paradigm by scaling both dataset diversity and evaluation breadth. Trained on 287,424 whole-slide images collected across multiple institutions and tissue types, PathOrchestra is evaluated on over 100 clinically relevant tasks, encompassing pan-cancer classification, lesion detection, biomarker assessment, and structured report generation [69]. Its results underscore a key insight: clinically meaningful performance in digital pathology is driven by large-scale, heterogeneous pre-training that captures real-world morphological variability.

Prov-GigaPath reinforces this conclusion by demonstrating that pretraining on provenance-aware, real-world datasets derived from large healthcare networks yields representations that generalize across patients, tissues, and clinical settings [70]. By explicitly addressing distributional shift and institutional variability, Prov-GigaPath highlights the importance of pathology-native foundation models for robust and deployable clinical AI systems.

Recently, PathologyVLM represents a state-of-the-art large vision–language model explicitly designed for pathology image understanding [71]. Trained on high-resolution whole-slide images paired with rich textual annotations, it captures multi-scale morphological features, spatial relationships, and contextual information crucial for histopathology and cytopathology interpretation. Unlike general-purpose multimodal models, PathologyVLM is optimized to align visual and textual representations, enabling clinicians to perform interactive queries, retrieve similar cases, and explore structured and semantically meaningful report drafts. Its integration within tool-augmented workflows allows it to complement pathology-native visual foundation models, providing interpretive and explanatory layers that enhance accessibility, reproducibility, and consistency without taking over diagnostic authority. In practice, PathologyVLM functions as a workflow-integrated copilot: it supports quality assurance, training, and report standardization, facilitates guideline-aligned documentation, and allows for real-time clinician interaction with complex image data. By leveraging a robust foundation in domain-specific data, PathologyVLM illustrates the emerging paradigm in computational pathology, where vision–language models extend the interpretive reach of validated visual encoders while ensuring that core diagnostic decisions remain with human experts and specialized foundation models.

##### Multimodal Copilots and Workflow Support in Pathology and Cytopathology

Within a foundation-model-centric framework, multimodal copilots are best understood as interface and coordination layers rather than autonomous diagnostic agents. Their primary function is to mediate interactions between clinicians and complex AI systems by translating model outputs into interpretable, structured, and clinically meaningful representations. LLMs pretrained on extensive clinical text corpora, such as those evaluated in the DRAGON benchmark, demonstrate strong performance in annotation, report drafting, clinical information extraction, and normalization of unstructured data, offering scalable and cost-effective support across multiple stages of the pathology workflow without supplanting human expertise or diagnostic responsibility [72].

Beyond efficiency gains, multimodal copilots contribute to standardization and consistency in reporting by enforcing structured templates, harmonizing terminology, and supporting guideline-aligned documentation. These capabilities are particularly relevant in high-throughput pathology settings, where variability in reporting practices and cognitive load can impact turnaround times and report quality. Importantly, the role of the copilot remains explicitly supportive: it facilitates information flow and interpretability while preserving human oversight and accountability.

In cytopathology, where annotated datasets are limited and morphological variability is high, maintaining a clear division of labor between visual inference and language-based interpretation is especially critical. Systems such as PathChat and TITAN exemplify this design philosophy by integrating large, pathology-native visual encoders with language models that summarize, contextualize, and explain model-derived findings rather than independently inferring diagnoses [65,66]. These systems enable natural language interaction with complex visual models, allowing clinicians to query results, explore alternative interpretations, and generate structured reports, while diagnostic reasoning remains anchored in specialized visual representations.

The importance of architecture-aware design is further highlighted by graph-based approaches such as GNNFormer, which explicitly model cell-to-cell relationships and spatial organization to capture biologically meaningful structures essential for accurate cytopathology reporting [66]. By incorporating inductive biases aligned with cellular organization, such models address limitations inherent to language-centric or loosely adapted multimodal systems. In this context, multimodal copilots act as translators between biologically grounded model outputs and clinical narratives, rather than as sources of independent diagnostic inference.

Collectively, these systems demonstrate that clinical value in digital pathology and cytopathology emerges from collaborative, human-in-the-loop architectures. Pathology-native visual models provide the diagnostic signal, pathology-adapted language components enhance interaction, explanation, and reporting, and multimodal copilots coordinate these elements within existing clinical workflows. This collaborative paradigm improves efficiency, report quality, and training while deliberately deferring diagnostic authority to validated models and human experts, in alignment with current best practices for responsible AI use in clinical research settings.

##### Model Adaptation, RAG, and Cost-Aware Strategies for Digital Cytology

The deployment of AI systems in digital pathology and cytology is shaped not only by algorithmic performance, but also by practical constraints, including computational cost, data availability, infrastructure heterogeneity, and the need for continual adaptation to evolving clinical knowledge and guidelines. These constraints are particularly relevant in cytopathology, where datasets are smaller, annotation is labor-intensive, and subspecialty-specific variability limits the feasibility of large-scale end-to-end model retraining.

Parameter-efficient fine-tuning techniques, such as Low-Rank Adaptation (LoRA) and Quantized LoRA (QLoRA), provide a practical solution. They enable adaptation of large language models to specific institutional, subspecialty, or workflow contexts with substantially reduced computational and memory overhead [72]. By modifying only a small subset of model parameters, these approaches allow customization of LLM behavior—for instance, reporting style, terminology alignment, or local guideline compliance—without requiring full model retraining. This ensures that LLMs support workflow integration without attempting primary diagnostic inference.

When combined with Retrieval-Augmented Generation (RAG), pathology-adapted LLMs can further address limitations related to static training data and knowledge obsolescence. RAG architectures allow LLMs to dynamically access curated, up-to-date, domain-specific knowledge, including clinical guidelines, consensus statements, exemplar cases, and institutional protocols, at inference time without altering the underlying diagnostic model [72,73]. This approach is particularly suitable for digital cytology, where large, curated datasets comparable to those in histopathology are often lacking.

In practice, RAG-enabled LLMs provide interpretive, educational, and reference-driven support rather than direct diagnostic inference. By delivering contextual knowledge alongside model outputs, they enhance clinician understanding, facilitate quality assurance, and support training and continuing education. Importantly, this design reinforces a central principle in computational pathology: LLMs augment human expertise and specialized visual models, while diagnostic authority remains with validated models and clinicians.

##### MedGemma and Domain-Specific LLMs

MedGemma exemplifies an emerging class of domain-specific large language models optimized for multimodal medical understanding. Built on the Gemma 3 architecture, its multimodal variants (4B and 27B) integrate a pretrained SigLIP image encoder capable of processing histopathology and other clinical images. MedGemma is Google’s medical LLM and serves as a proof-of-concept system; it is not clinically approved. This enables the model to support image interpretation, structured report generation, and clinically grounded reasoning within a unified framework [74,75,76,77]. While not clinically approved, MedGemma serves as a proof of concept for how medical-specific pretraining and architectural alignment can enhance workflow integration without replacing human expertise or validated diagnostic models.

From a systems perspective, MedGemma-like models are best understood as pathology-adapted language components within tool-augmented pipelines rather than standalone diagnostic engines. When integrated with pathology-native visual foundation models, such as CONCH, PathOrchestra, or TITAN, these LLMs [65,66,67,68,69] can:

- Contextualize visual model outputs to improve interpretability;
- Generate structured and standardized reports aligned with clinical guidelines;
- Facilitate interactive exploration of findings for clinicians.

All diagnostic inference remains grounded in validated visual foundation models, ensuring that LLM outputs augment rather than supplant clinical decision-making.

This layered architecture—combining pathology-native foundation models, pathology-adapted LLMs, and cost-aware adaptation strategies (e.g., LoRA/QLoRA fine-tuning)—preserves diagnostic rigor while leveraging the strengths of LLMs in reasoning, communication, and workflow integration. By explicitly separating diagnostic signal extraction from interpretive and narrative functions, these pipelines maintain reproducibility, traceability, and accountability, aligning with emerging regulatory, ethical, and safety expectations for responsible clinical AI deployment in high-stakes domains such as digital pathology and cytopathology.

Finally, RAG further extends the utility of these pipelines by allowing LLMs to access curated, up-to-date, domain-specific information—such as clinical guidelines, exemplar cases, and institutional protocols—without modifying the underlying diagnostic model. In practice, RAG-enabled LLMs provide interpretive, educational, and reference-driven support, enhancing clinician understanding, quality assurance, and training, while diagnostic authority firmly remains with validated pathology models and human experts [72,73]. Smaller, task-specific neural networks can be incorporated as tools to further strengthen domain-specific capabilities, supporting a modular and collaborative AI workflow.

Table 6 provides a structured overview of the studies that informed our analysis of large language models (LLMs), vision–language models (VLMs), and domain-specific foundation models in digital pathology and cytopathology. The table is organized to summarize each study’s technological approach, focus, and observed boundaries, highlighting their role as supportive tools rather than standalone diagnostic systems. This organization emphasizes the distinction between visual foundation models, language components, and tool-augmented pipelines, illustrating how clinical, interpretive, and workflow functions are distributed across these systems. Web-based resources, such as MedGemma, are included to reflect practical examples of workflow-integrated multimodal LLMs.

## 4. Discussion

### 4.1. Discussion—Key Findings and Responses to Research Questions

This review was structured around five key questions: (1) How are immersive technologies being applied in healthcare and cytopathology, and what benefits do they provide for training, diagnostics, and workflow optimization? (2) In what ways can VLM-based copilots support clinical decision-making, report generation, and cognitive augmentation in cytopathology? (3) What technical, operational, and workflow challenges must be addressed to integrate these tools into routine practice? (4) How do ethical, regulatory, and organizational factors influence their safe and effective adoption? (5) How is the role of the cytopathologist evolving in response to immersive tools and AI copilots, and what implications emerge for professional competencies and training?

The literature indicates that immersive technologies, such as virtual and augmented reality platforms, can enhance procedural training, spatial understanding, and collaborative learning (Clay et al., 2024 [52]; Chance, 2025 [53]; Lim & Yap, 2024 [54]; Iqbal et al., 2024 [55]). They allow learners to rehearse clinical procedures in controlled environments, which is particularly valuable in cytopathology, where real patient exposure is limited. However, benefits are often contextual and evidence remains limited: studies frequently involve small cohorts, simulated scenarios, or early-stage prototypes, which restricts generalizability. High-quality empirical validation in real-world clinical workflows is still lacking, and the effectiveness of immersive technologies depends heavily on system fidelity, user training, and integration with standard clinical processes. These observations partially address the first research question, highlighting both potential and limitations.

For VLM-based copilots, evidence shows that these systems can support clinical decision-making, interactive report generation, and structured interpretation of complex data (Lu et al., 2024 [64,68]; Ding et al., 2024 [65]; Yan et al., 2025 [69]; Xu et al., 2024 [70]). They function as augmentative tools rather than autonomous diagnostic agents, providing contextualized suggestions, evidence synthesis, and interactive guidance. Yet, performance is highly dependent on the quality of visual encoders, the specificity of pretraining datasets, and the design of task-specific pipelines. Risks include reduced reliability with rare entities, sensitivity to input prompts, and potential overreliance by users (Liu et al., 2024 [67]; Al-Asi et al., 2025 [73]). This nuanced understanding directly responds to the second research question, illustrating how AI can enhance cognitive processes without replacing human expertise, while also delineating boundaries of applicability.

Integration into clinical practice faces multiple challenges. Technical constraints include variability in hardware, software, and platform maturity, as well as interoperability with existing laboratory information systems (Dai et al., 2025 [71]; Al-Asi et al., 2025 [73]). Operational challenges involve user training, workflow adaptation, and balancing cognitive load. Organizational, regulatory, and ethical considerations impose additional limitations: clear accountability, adherence to clinical guidelines, and structured human oversight are essential to mitigate risks of misuse (Jia Li et al., 2025 [61]; Kaiser et al., 2025 [62]; Eriksen et al., 2024 [63]). These points address the third and fourth research questions, emphasizing that successful adoption requires careful planning, validation, and governance.

Finally, the role of the cytopathologist is evolving in response to these technologies. Professionals are expected to integrate AI copilots and immersive tools into their practice, which may shift required competencies toward digital literacy, data interpretation, and workflow management, while maintaining diagnostic expertise (Zhou et al., 2023 [66]; Liu et al., 2024 [67]). This observation responds to the fifth research question, suggesting that while these technologies provide augmentation, they also reshape professional identity, training needs, and the division of responsibilities within the cytopathology team.

Overall, the evidence presents a balanced picture: immersive technologies and VLM-based copilots offer measurable enhancements in training, workflow, and cognitive support, but their benefits are bounded by technical, operational, and organizational constraints. They are not yet mature for autonomous diagnostic use, and careful integration with validated clinical models and human expertise remains essential. These findings set the stage for a more detailed examination in the subsequent sections of the discussion: *Technological Capabilities and Maturity, Workflow and Educational Applications, Professional Implications, and Conceptual Limitations and Responsible Use*. The following sections of the discussion are further corroborated by additional evidence from the literature [78,79,80,81,82,83,84,85,86,87,88,89,90,91,92,93,94,95,96,97,98,99,100,101,102,103,104], providing support for the analysis of technological capabilities, workflow applications, professional implications, and ethical considerations.

### 4.2. Discussion: Conceptual and Practical Considerations for Immersive and AI-Assisted Systems in Cytopathology

#### 4.2.1. Technological Capabilities and Maturity

The reviewed literature suggests that immersive technologies and AI-based copilots in healthcare and cytopathology are characterized by heterogeneous levels of technological maturity. Virtual and augmented reality systems have reached sufficient stability for use in training and educational contexts, where controlled environments and predefined scenarios limit clinical risk (Clay et al., 2024 [52]; Lim & Yap, 2024 [54]; Iqbal et al., 2024 [55]). In these settings, immersive platforms provide high-fidelity simulations that support procedural rehearsal, spatial understanding, and exposure to rare or complex scenarios. However, their translation into routine diagnostic workflows remains limited by hardware costs, infrastructure requirements, and variability in platform performance.

In parallel, advances in computational pathology have been driven primarily by domain-specific visual foundation models rather than by general-purpose LLMs or VLMs. Models such as CONCH, PathOrchestra, and Prov-GigaPath demonstrate that diagnostic performance in digital pathology relies on large-scale, pathology-native pretraining capable of capturing morphological variability across tissues and institutions (Lu et al., 2024 [68]; Yan et al., 2025 [69]; Xu et al., 2024 [70]). Vision–language components, when present, act as interpretive or interactive layers rather than primary sources of diagnostic inference. Pathology-adapted VLMs further extend accessibility through multimodal retrieval and navigation but remain constrained by the quality and specialization of their visual encoders (Dai et al., 2025 [71]).

Overall, the current technological landscape supports augmentation and workflow assistance, but not autonomous diagnostic deployment. Maturity varies across components: while immersive hardware and foundation models are increasingly robust, their integration into cohesive, validated clinical systems is still evolving.

#### 4.2.2. The Copilot Paradigm: Human-in-the-Loop, Trends, and Lessons from Other Diagnostics Fields

Recent bibliometric and narrative trends indicate that the concept of the AI copilot has emerged as a central organizing principle in biomedical artificial intelligence. Searches in PubMed reveal a rapidly expanding body of literature, particularly regarding immersive technologies, up to the period during which this study was conducted. A simple search using the terms.

*(“Virtual reality”[Title/Abstract]) OR (“Extended reality”[Title/Abstract]) OR (“Augmented reality”[Title/Abstract])*

Demonstrates a total of 31,462 studies [97] since 1991, including 5521 reviews (18%). Among these, 25,362 studies were published in the last 10 years, and 18,536 studies in the last 5 years (59%), highlighting the accelerating adoption of immersive approaches in biomedical research.

Large language models (LLMs), as expected, are of more recent diffusion. A PubMed search using *“large language model”[Title/Abstract]* retrieved 3901 [98] studies, including 323 reviews (8%). Except for a single study published in 2019, all studies have been concentrated within the last three years, reflecting the rapid rise of LLM-based research in biomedicine. The relatively low proportion of review articles among LLM studies indicates that the field is still in an early, exploratory phase. Most publications focus on original research, model development, or preliminary applications, rather than on synthesis, guidelines, or consolidated evidence. This contrasts with immersive technologies, where the higher number of reviews reflects a more mature body of literature with established conceptual frameworks, methodological consensus, and educational or clinical integration.

The overall literature on immersive technologies and large language models (LLMs) in biomedicine has grown rapidly over the last decade. PubMed searches and bibliometric analyses show accelerating publication rates across original studies, preprints, clinical guidelines, and related databases. However, when the focus is narrowed to cytopathology, the number of studies is extremely limited—so few that they can be “counted on the fingers of one hand.” as reported in Section 2. This scarcity highlights the early stage of empirical research in this domain and motivated the implementation of a CENR, aimed at synthesizing the available evidence from different sources and framing it within a coherent conceptual structure. The purpose is to reaffirm the copilot paradigm: AI systems should be understood as cognitive assistants, supporting and structuring human decision-making, rather than as autonomous diagnostic agents.

To contextualize the copilot concept in cytopathology, it is useful to examine more mature diagnostic fields such as radiology. In radiology, AI systems have evolved from stand-alone classifiers into integrated copilots that actively support multiple stages of clinical workflows, including image triage, lesion detection, automated measurements, report drafting, guideline-aligned decision support, professional training, and even patient-facing applications, such as immersive interventions that reduce anxiety, improve patient understanding, and foster collaborative engagement [99,100,101,102]. Across these applications, diagnostic responsibility remains with the human expert, and emerging regulatory frameworks consistently reinforce that AI should function as a supportive cognitive agent, rather than as an autonomous decision-maker. Workflows are designed so that algorithms propose, rank, or visualize hypotheses, while the clinician interprets, validates, and finalizes decisions.

The value of AI copilots lies not in providing epistemic authority, but in mediating between heterogeneous data sources, complex clinical guidelines, and human reasoning. When combined with immersive or interactive visualization, these copilots allow clinicians to navigate, interrogate, and contextualize high-dimensional biomedical information—capabilities that exceed what static interfaces or isolated tools can achieve [101,102]. Reference [103] directly reinforces this perspective by examining the role of image biomarkers and explainable AI: the study compares handcrafted features versus deep-learned features and highlights how interpretability of AI-derived representations is critical to supporting human understanding, trust, and validation in diagnostic decision-making. This underscores that effective copilot systems require both computational accuracy and transparent feature representation, enabling clinicians to trace AI reasoning and integrate it safely into their workflow.

In cytopathology, the limited availability of copilot-oriented studies does not indicate a lack of clinical relevance; rather, it reflects the absence of a well-defined conceptual and structural framework that connects large language models (LLMs), pathology-native visual foundation models, and immersive interfaces to practical diagnostic workflows. Unlike radiology, which has successfully transitioned from “AI as a tool” to “AI as a copilot” [101,102], cytology remains largely fragmented, with most AI applications evaluated in isolated, narrowly scoped tasks. This fragmentation explains why purely quantitative or systematic syntheses are insufficient to capture the potential of AI-assisted workflows in cytopathology.

By grounding this review in both bibliometric trends and the more mature radiology copilot paradigm, the CENR highlights how human-in-the-loop, immersive, and AI-assisted systems could evolve in cytopathology from isolated diagnostic tools into fully integrated copilots. The focus is on workflow integration, interpretability, and human augmentation, rather than on individual model performance. Incorporating insights from [103] further strengthens this approach by demonstrating that explainable features—whether handcrafted or deep-learned—are essential for clinicians to understand, validate, and trust AI outputs, ensuring safe and effective adoption in high-stakes diagnostic environments. This conceptual framework can guide future research, workflow development, educational programs, and patient engagement strategies, providing a roadmap for translating isolated AI tools into clinically meaningful copilots in cytopathology.

#### 4.2.3. Workflow and Educational Applications

Across the reviewed studies, immersive technologies are most consistently applied in education, training, and workflow simulation, where they offer clear advantages with relatively low risk. VR and AR platforms enable repeated practice, visualization of complex anatomical or cellular structures, and remote collaboration, addressing long-standing limitations in cytopathology training related to case availability and variability (Clay et al., 2024 [52]; Venkatesan et al., 2021 [59]). These benefits align with broader trends in medical education, where immersive tools complement traditional instruction rather than replacing hands-on experience.

VLM-based copilots contribute to workflow support by assisting with report drafting, information retrieval, and interactive exploration of model outputs (Ding et al., 2024 [65]; Liu et al., 2024 [67]). In high-throughput pathology settings, such tools may reduce cognitive load by structuring information, harmonizing terminology, and supporting guideline-aligned documentation (Bosma et al., 2025 [72]). However, evidence of direct improvements in diagnostic accuracy or efficiency remains limited and context-dependent. Integration challenges include interoperability with laboratory information systems, adaptation to local reporting practices, and the need for sustained user training.

Thus, while workflow and educational applications represent the most mature and immediately applicable use cases, their effectiveness depends on careful alignment with existing clinical processes and realistic expectations regarding their capabilities.

#### 4.2.4. Professional Implications and the Evolving Role of the Cytopathologist

The introduction of immersive tools and AI copilots is reshaping the professional landscape of cytopathology, not by displacing expertise, but by reconfiguring how expertise is exercised. Cytopathologists are increasingly positioned as supervisors of AI-assisted workflows, responsible for interpreting outputs, validating findings, and contextualizing results within clinical narratives (Zhou et al., 2023 [66]; Liu et al., 2024 [67]). This shift emphasizes competencies in digital literacy, critical appraisal of AI outputs, and integration of multimodal information.

At the same time, the literature cautions against overestimating the autonomy of AI systems. Evaluations of LLM performance in examination-style or simulated tasks demonstrate strong knowledge recall but also reveal vulnerabilities to hallucination and framing effects [63]. These findings reinforce the importance of maintaining human oversight and professional accountability. Rather than eroding professional identity, immersive technologies and copilots may strengthen the cytopathologist’s role as an integrator of complex information and a guarantor of diagnostic rigor, provided that training and governance structures evolve accordingly.

#### 4.2.5. Conceptual Limitations and Responsible Use of LLMs and Copilots

A consistent theme across the reviewed literature is the need to frame LLMs and VLM-based copilots as supportive, augmentative tools, not autonomous diagnostic agents. Clinical decision-support studies explicitly position LLMs as mechanisms for synthesizing guidelines, structuring information, and supporting reasoning, while rejecting their use as independent diagnostic authorities (Jia Li et al., 2025 [61]; Kaiser et al., 2025 [62]). This distinction is particularly critical in cytopathology, where diagnosis depends on fine-grained morphological and spatial cues that are not reliably captured by general-purpose language models.

Tool-augmented architectures, including the integration of LLMs with domain-specific visual models and smaller task-focused networks, represent a promising direction for responsible deployment (Lu et al., 2024 [68]; Ding et al., 2024 [65]). Retrieval-Augmented Generation (RAG) further mitigates limitations related to static knowledge and hallucination by grounding outputs in curated, up-to-date sources (Al-Asi et al., 2025 [73]). Domain-specific LLMs such as MedGemma illustrate how medical pretraining can improve alignment with clinical workflows, but these systems remain proof-of-concept and are not clinically approved for diagnostic use [74,75,76,77].

From a regulatory and ethical perspective, the probabilistic nature of LLMs, their sensitivity to input framing, and their lack of intrinsic biological grounding necessitate clear governance, validation, and accountability frameworks. Responsible adoption therefore depends on preserving a strict separation between diagnostic inference—performed by validated, pathology-native models and human experts—and interpretive, communicative, or educational functions supported by language-based systems.

### 4.3. Discussion: Implications for Clinical Practice, Education, and Governance of Immersive and Copilot Technologies

#### 4.3.1. Recommendations for Clinical Practice and Education

Evidence from the reviewed literature indicates that immersive technologies and AI-based copilots currently offer selective and supportive contributions, rather than comprehensive or transformative solutions. Their applicability varies substantially depending on technological maturity, institutional readiness, and the specific clinical or educational task.

With respect to immersive technologies, studies on virtual histology and three-dimensional tissue visualization primarily emphasize their value in educational and exploratory contexts. Liimatainen et al. demonstrated that VR-based multi-scale visualization can facilitate interactive exploration of tissue architecture, particularly for understanding spatial relationships that are difficult to appreciate on conventional two-dimensional slides [80]. However, this evidence remains largely preclinical and exploratory, and significant technical overheads related to data handling and rendering complexity limit immediate clinical translation. Similarly, systematic analyses of virtual histopathology methods underline that these approaches remain largely experimental, with heterogeneous validation strategies and limited evidence of diagnostic equivalence to standard histopathology workflows [79].

In contrast, the literature on digital pathology infrastructure—including whole-slide imaging, image compression, and standardization—provides more concrete guidance for clinical implementation. Garcia-Rojo et al. emphasized the importance of DICOM standardization for WSI to ensure interoperability and long-term sustainability [81], while Zarella and Jakubowski showed that optimized video compression is a practical enabler for extending WSI into cytology without compromising diagnostic usability [82]. Longitudinal institutional experiences further suggest that gradual integration of cytology into digital pathology workflows is feasible, but requires sustained organizational effort, training, and iterative workflow redesign [83,84].

From an educational perspective, immersive and simulation-based tools appear most defensible as adjuncts to traditional training. Studies on immersive teaching environments and AI-supported simulation systems report improvements in engagement and procedural confidence, particularly in early training phases [91,92]. Nonetheless, these benefits are primarily pedagogical, and evidence supporting direct transfer to diagnostic accuracy in cytopathology remains limited. Consequently, immersive technologies are best positioned as complementary educational resources, rather than replacements for microscope-based training or supervised clinical practice.

Regarding LLM- and VLM-based copilots, the literature consistently frames these systems as tools for cognitive and administrative support, not autonomous diagnostic agents. Narrative and scoping reviews in pathology and cytopathology describe applications such as report drafting, structured documentation, literature retrieval, and educational assistance [78,95]. It is important to note that most evidence is still descriptive or preclinical, highlighting persistent challenges related to hallucinations, lack of domain grounding, and regulatory uncertainty [96]. This reinforces the need to constrain LLM use to non-diagnostic functions within clinical workflows.

Earlier work on deep learning in cytology further contextualizes these limitations. McAlpine et al. documented structural challenges in developing reliable AI systems for cytology, including dataset scarcity, variability in specimen preparation, and annotation burden [85]. These constraints underscore why language-based systems—despite their versatility—cannot substitute for validated image-based models or expert judgment in diagnostic decision-making.

#### 4.3.2. Future Directions for Immersive and Copilot Technologies in Cytopathology

Future research trajectories point toward integration rather than replacement. For immersive technologies, the most promising directions involve tighter coupling with established digital pathology platforms, enabling selective use of 3D visualization or simulation where added value is clear—such as training, multidisciplinary communication, or complex case review—rather than routine diagnostics [79,80,90].

In parallel, emerging educational applications, including AI-generated feedback and simulation-based deliberate practice, suggest potential roles in cytopathology training programs, particularly for procedural skills and rapid on-site evaluation (ROSE) scenarios [91,92,93,94]. However, rigorous educational and clinical validation is still required, as most current evidence derives from pilot or preclinical studies.

For AI copilots, future developments increasingly emphasize tool-augmented architectures, such as retrieval-augmented generation (RAG) and integration with domain-specific models. Reviews focusing on LLMs in pathology suggest that such hybrid systems may improve contextual reliability while preserving human oversight [95,96]. Conceptual discussions around specialized models—such as MedGemma and related domain-adapted networks—illustrate potential pathways for safer deployment, but current evidence remains largely descriptive and preclinical, relying on organizational or technical reports rather than peer-reviewed clinical validation [74,75,76,77].

#### 4.3.3. Broader Outlook: Ethical, Regulatory, and Organizational Considerations

Across both immersive and AI-assisted technologies, ethical, legal, and organizational factors are central constraints rather than secondary considerations. Security and privacy analyses of virtual reality environments identify vulnerabilities related to data exposure, authentication, and system integrity, which are particularly relevant when patient-derived data are involved [86,87]. Regulatory-oriented studies stress the importance of applying medical device standards and risk management frameworks to immersive systems, especially in remote or hybrid care settings [88].

Ethical analyses of virtual agents and immersive healthcare technologies highlight automation bias, opacity, and role ambiguity, reinforcing the need for clear accountability structures and professional oversight [89]. Organizational experiences with digital pathology adoption confirm that technological feasibility alone is insufficient; success depends on governance, training, and alignment with institutional workflows and professional culture [83,84].

Taken together, the literature supports a cautious and incremental adoption model, in which immersive technologies and AI copilots are framed as augmentative tools that enhance—rather than redefine—the role of the cytopathologist. This perspective aligns with current regulatory expectations and provides a realistic foundation for future preclinical and clinical experimentation and educational innovation.

## 5. Conceptual Synthesis and Future-Oriented Framework

The preceding sections have highlighted both the potential and the current limitations of immersive technologies and AI-assisted systems in cytopathology. Rather than converging toward a single dominant solution, the literature reveals a fragmented landscape of tools, proof-of-concept studies, and narrowly scoped applications, often evaluated in isolation and with limited clinical validation. This fragmentation complicates systematic comparison and makes it difficult to translate individual technical advances into coherent, clinically meaningful workflows.

For this reason, the aim of this final section is not to introduce new empirical results, but to provide a conceptual synthesis that connects the main findings of the review into an integrated and forward-looking framework. By combining representative visual examples, workflow analysis, and schematic modeling, this section addresses two complementary needs: (i) making explicit the intrinsic visual and cognitive challenges of cytopathological practice, and (ii) illustrating how emerging copilot-oriented architectures—integrating visual foundation models, language-based reasoning layers, and immersive or interactive interfaces—could be structured in a clinically responsible manner.

In line with the copilot paradigm discussed earlier, this synthesis explicitly maintains the cytopathologist as the final decision-maker. AI and immersive technologies are framed as supportive layers that enhance perception, organization, and interpretation of complex information, rather than as autonomous diagnostic agents. The proposed framework therefore emphasizes human-in-the-loop control, interpretability, and workflow alignment, reflecting both current regulatory expectations and lessons learned from more mature diagnostic domains.

The following subsections operationalize this synthesis. First, representative cytopathology images are used to illustrate common sources of visual ambiguity and cognitive load that motivate the need for advanced visualization and assistance tools. Second, a schematic workflow is proposed to map how copilot-oriented systems—augmented by VLM-based interaction and immersive visualization—could evolve from isolated tools into integrated components of future cytopathology practice.

### 5.1. Visual and Workflow Challenges in Cytopathology: Representative Examples

Cytopathology is inherently challenging due to the fine-grained morphology of individual cells, heterogeneous sample quality, and the presence of background artifacts. Among the most widely applied domains in clinical practice are cervical cytology and lung cytology, which together illustrate the interpretive demands that motivate the adoption of immersive technologies and AI-assisted copilots [104].

**Figure 1:**
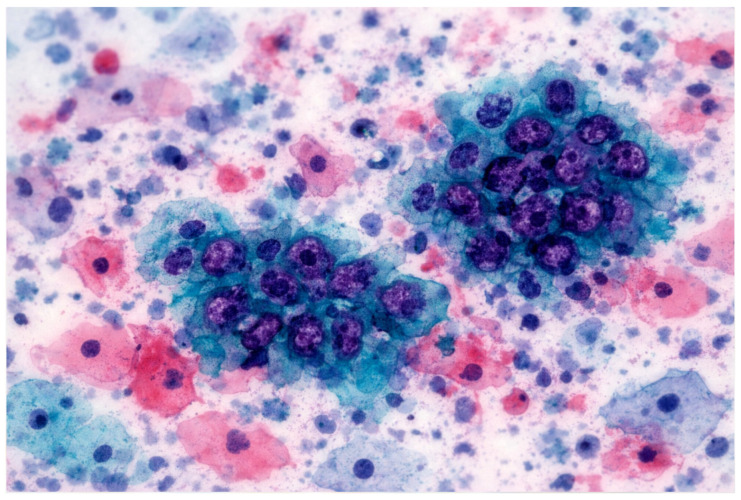
Cervical Cytology—Pap Smear. The image depicts a cervical cytology stylized specimen stained with the Papanicolaou technique. Two clusters of cells show large, irregular, dark-staining nuclei characteristic of malignancy, while surrounding normal cells are smaller with regular nuclei and lighter cytoplasm. Cellular debris and occasional red blood cells are visible. This representative example illustrates the interpretive challenges faced by cytopathologists in identifying subtle dysplastic changes and highlights the potential utility of immersive XR platforms and AI-assisted copilots to enhance visualization, annotation, and report generation [52,53,54,55,56,58,78].

Cervical cytology (Figure 1), typically stained using the Papanicolaou (Pap) technique, allows for the detection of pre-cancerous and cancerous changes. In the presented image, two clusters of cells display large, irregular, dark-staining nuclei characteristic of malignant cells. The cytoplasm appears blue–green, while surrounding normal cells are smaller with regular nuclei and lighter staining. Cellular debris and occasional red blood cells are also present.

The cytopathologist plays a central role: examining nuclear morphology, assessing nuclear-to-cytoplasmic ratios, identifying dysplastic or malignant clusters, and issuing a standardized cytology report (e.g., Bethesda System) [56,58,82,83].

This work is not without challenges. Early cellular changes can be subtle, requiring careful observation. Distinguishing atypical cells from artifacts or reactive changes is crucial to avoid false positives or negatives. Malignant cells may be isolated or in small clusters, increasing interpretive difficulty. High concentration, advanced microscopy skills, and ongoing training are essential to maintain accuracy.

**Figure 2:**
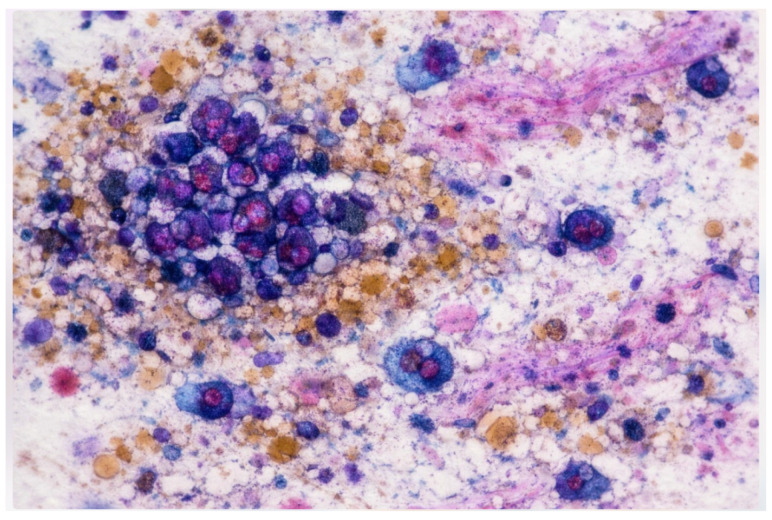
Lung Cytology—Sputum/Bronchial Washing The image shows a stylized lung cytology specimen obtained from sputum or bronchial washings. Malignant cells appear as isolated cells or small clusters with irregular, hyperchromatic nuclei and prominent nucleoli, embedded within a background of mucus, inflammatory cells, and cellular debris. Accurate identification requires careful scanning and contextual interpretation. This representative example highlights the diagnostic complexity of lung cytology and illustrates the potential role of XR and VLM-based tools in supporting multi-scale visualization, annotation, and structured reporting [52,53,54,55,56,58,78].

Lung cytology (Figure 2), obtained from sputum or bronchial washings, presents complementary diagnostic challenges. Malignant cells may appear as isolated cells or small clusters, with irregular, hyperchromatic nuclei and prominent nucleoli. Cytoplasm varies in staining intensity, while mucus, inflammatory cells, and cellular debris in the background can obscure critical features. The cytopathologist must carefully integrate morphological findings with clinical context, assessing nuclear morphology, cytoplasmic features, and subtle atypia to provide an accurate report [56,58,82,83].

Challenges include heterogeneous cell populations, background material, and subtle cellular changes that can mimic reactive or inflammatory processes. Detecting sparse malignant cells requires meticulous scanning and interpretive expertise, highlighting the importance of training and cognitive support. Extended reality (XR) technologies—including virtual and augmented reality—can assist by providing immersive slide navigation, multi-scale exploration, and interactive annotation, reducing cognitive load and improving spatial understanding [52,53,54,55,59,79,80,91,92].

Together, cervical and lung cytology exemplify the visual and cognitive complexity of cytopathology and serve as paradigmatic examples for the integration of immersive technologies and AI copilots. By allowing interactive exploration, pattern highlighting, and context-aware suggestions, these tools can support cytopathologists in both education and routine practice. As reviewed by Lastrucci et al. (2024) [78], these applications are the most extensively studied in AI-assisted cytology, providing a practical context to evaluate the impact of such technologies on workflow and diagnostic quality.

Extended reality (XR) technologies—including virtual and augmented reality—offer significant potential to support the diagnostic challenges highlighted in cervical and lung cytology (Figure 1 and Figure 2) [52,53,54,55,59,79,80,91,92]. XR platforms enable immersive navigation of cytology slides, allowing cytopathologists to explore samples at multiple scales, highlight regions of interest, and annotate interactively. By providing a three-dimensional, manipulable view of cellular structures, XR enhances spatial understanding and perceptual accuracy, reducing cognitive load and mitigating the risk of overlooking subtle malignant changes. Repeated practice within a controlled XR environment allows cytopathologists to refine interpretive skills, while collaborative review features enable remote consultation and training. Exposure to rare or complex cases, which may be encountered infrequently in routine practice, becomes feasible without compromising patient safety. Importantly, XR preserves the cytopathologist as the ultimate decision-maker, ensuring that clinical authority remains firmly in human hands. A comparative synthesis of cytopathologist tasks, diagnostic challenges, and the potential contributions of XR technologies and AI-based copilots in cervical and lung cytology is provided in Table 7.

Vision–language models (VLMs) complement XR by translating visual information into structured, interpretable outputs [64,65,66,67,68,69,70,71,78,95,96]. These models can identify clusters of atypical cells, extract morphometric and spatial features, and generate draft reports that align with clinical guidelines, assisting with differential diagnosis framing and documentation. VLM-based copilots can highlight salient patterns, suggest potential interpretations, and cross-reference curated literature, streamlining the reporting workflow and supporting less experienced cytopathologists. Crucially, these AI tools are augmentative rather than autonomous, designed to enhance human cognition without replacing expert judgment [61,62,63,85].

When combined, XR and VLMs create a synergistic environment that strengthens both training and workflow efficiency. XR ensures accurate, immersive perception of complex cellular morphology through multi-scale navigation, interactive annotation, and spatial exploration, reducing cognitive load and enhancing visual comprehension [52,53,54,55,59,79,80,91,92]. In parallel, VLMs provide interpretive scaffolding, documentation support, and context-aware guidance. By connecting image visualization, AI-driven feature extraction, and language-based summarization into an integrated workflow, such systems can improve diagnostic throughput, reduce fatigue, support continuous learning, and maintain interpretability and professional accountability, addressing both educational and clinical needs within cytopathology.

### 5.2. From Isolated Tools to Copilot-Oriented Workflows: A Conceptual Roadmap

The cervical and lung cytology examples discussed in Section 5.1 illustrate how cytopathology combines fine-grained visual interpretation with heterogeneous digital inputs and high cognitive demands. In current clinical practice, whole-slide images, digital viewers, AI-based image analysis tools, reporting systems, and reference guidelines are often accessed as separate components. This fragmentation requires cytopathologists to manually integrate visual findings, clinical context, and documentation, and has been identified as a key limitation of contemporary digital pathology and cytology workflows [57,83,84,90].

**Figure 3:**
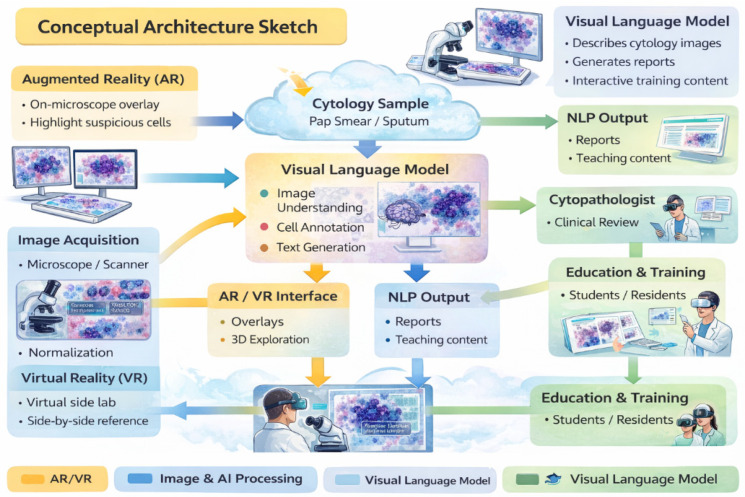
Conceptual Framework for Digital Cytology. This schematic illustrates the modular structure of a digital cytology workflow, showing isolated tools and their interactions. Layers include slide acquisition and management, digital visualization, and extended reality (XR) platforms for immersive navigation and multi-scale exploration. AI-based perception modules, including vision–language models (VLMs), operate in parallel to extract features, highlight regions of interest, and assist in structured reporting. Arrows indicate data flow between layers, demonstrating how human interpretation remains central while digital tools enhance efficiency, perceptual accuracy, and documentation. This conceptual framework highlights the potential for integration into copilot-oriented workflows, providing context for the diagnostic scenarios illustrated in Figure 1 and Figure 2.

Figure 3 conceptualizes a transition from isolated tools toward an integrated, copilot-oriented workflow. At the foundational level, digital cytology slides and associated metadata are acquired and managed through standardized digital pathology infrastructures [56,57,81]. Extended reality (XR) technologies operate primarily at the interface layer, providing immersive slide navigation, multi-scale exploration, and interactive annotation. These capabilities have been shown to reduce cognitive load, enhance spatial understanding, and support repeated practice, collaborative review, and exposure to rare or complex cases in medical education and image-based disciplines [52,53,54,55,59,79,80,91,92].

Building on this visual interface, AI-based perception modules—including pathology foundation models and vision–language models (VLMs)—support automated feature extraction, region prioritization, and structured representation of visual findings [64,65,66,67,68,69,70,71]. VLMs further translate visual information into natural-language summaries and draft reports aligned with clinical guidelines, assisting with differential diagnosis framing and documentation [78,95,96]. Importantly, these systems are designed as assistive copilots rather than autonomous decision-makers, reinforcing human expertise and preserving interpretability, accountability, and professional responsibility [61,62,63,85].

**Figure 4:**
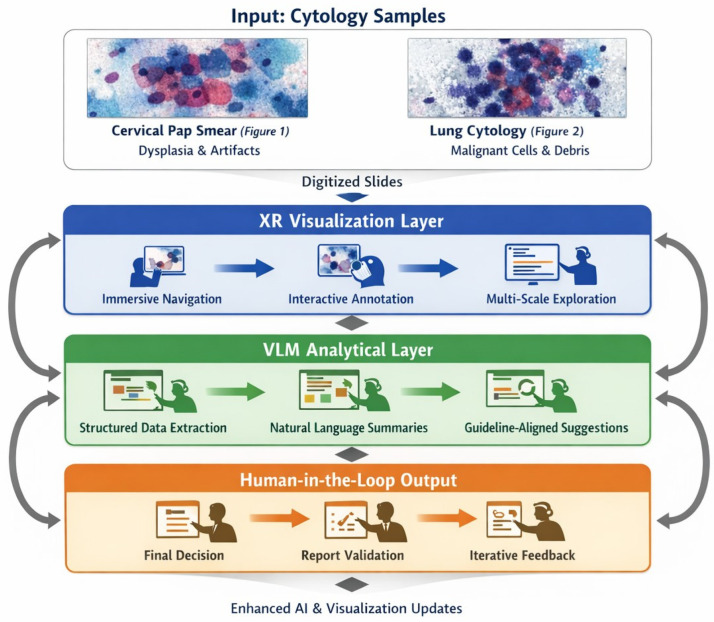
Copilot-Oriented Cytopathology Workflow. This workflow diagram represents a copilot-oriented approach in cytopathology, integrating XR-assisted visualization, AI feature extraction, and VLM-supported report generation into a continuous interpretive loop. Inputs from digital slides, patient metadata, and prior cases are combined in interactive layers. XR tools enable multi-scale slide navigation, highlighting subtle or sparse cellular features, while VLMs organize observations into guideline-aligned summaries and assist differential diagnosis. Bidirectional arrows emphasize iterative human–AI collaboration, linking visual exploration, pattern recognition, and structured documentation. This figure connects abstract workflow design with real-world applications in cervical (Figure 1) and lung cytology (Figure 2), illustrating the educational and clinical potential of copilot integration.

Figure 4 links this abstract architecture to the concrete diagnostic scenarios introduced in Figure 1 (cervical cytology) and Figure 2 (lung cytology). In cervical cytology, XR-supported visualization facilitates the identification of subtle dysplastic changes and small malignant clusters, while VLM-based copilots assist in organizing observations according to standardized reporting systems such as the Bethesda classification. In lung cytology, immersive multi-scale navigation supports the detection of sparse malignant cells within heterogeneous backgrounds, and language-based models help structure findings and integrate clinical context. In both cases, visual exploration, AI-assisted pattern recognition, and language-based synthesis interact continuously during cytopathological reasoning rather than functioning as isolated steps.

Together, Figure 3 and Figure 4 outline a progressive and incremental integration strategy. Early adoption is most appropriate in low-risk contexts such as education, simulation, case review, and report drafting, where immersive visualization and language-based assistance can enhance learning, consistency, and efficiency without influencing diagnostic authority [52,53,91,92]. As digital pathology infrastructures mature and interoperability improves [57,81,83], selected copilot functions—such as guided navigation, prioritization of suspicious regions, and guideline-aligned documentation—may progressively extend into selected routine tasks, while preserving the cytopathologist as the final arbiter of diagnosis.

In this copilot-oriented framework, XR primarily enhances perception, spatial understanding, and engagement with complex cellular morphology, whereas VLMs provide interpretive scaffolding, documentation support, and context-aware guidance. Their integration directly addresses the visual and cognitive challenges exemplified by cervical and lung cytology in Section 5.1, offering a scalable and clinically grounded roadmap for future cytopathology workflows.

## 6. Limitations and Future Direction

The present Conceptual Exploratory Narrative Review (CENR) was conducted under conditions where alternative approaches were largely impractical. The field of immersive technologies and Vision–Language Model (VLM)-based copilots in cytopathology is extremely novel and rapidly evolving, with very limited peer-reviewed empirical evidence. Traditional systematic review methodologies were not feasible because standard bibliographic databases returned sparse results, and most early implementations exist only in preprints, gray literature, technical reports, or pilot projects. In this context, the CENR approach allowed a pragmatic and integrative exploration of emerging applications, trends, and conceptual patterns without imposing the strict inclusion and exclusion criteria typical of systematic reviews. The inclusion of preprints is justified, as they provide early visibility into experimental implementations, pilot projects, and ongoing discussions that are otherwise unavailable in conventional journals.

Nonetheless, this approach has inherent limitations. First, it is not systematic, so the findings do not provide exhaustive coverage or quantitative synthesis. The inclusion of preprints and gray literature increases the potential for selection bias, as these sources may not have undergone formal peer review.

Second, the review is narrative and interpretive, emphasizing conceptual understanding, emerging patterns, and potential applications rather than definitive empirical outcomes. The evidence base is limited both in size and methodological rigor, reflecting the early-stage nature of the field.

Third, the review draws on heterogeneous sources across multiple disciplines—including computer science, medical informatics, digital pathology, education, and human–technology interaction. As such, insights should be regarded as illustrative and exploratory, highlighting potential opportunities and conceptual linkages rather than conclusive findings.

Finally, while the review identifies promising applications of XR and VLM technologies, the real-world impact on routine cytopathology practice remains largely theoretical. Issues such as usability, workflow integration, diagnostic reliability, and educational efficacy will need empirical validation in future studies.

Despite these limitations, our hope is that the CENR provides a framework for understanding early-stage developments, identifies key conceptual dimensions, and highlights areas where XR and VLM-based copilots may augment training, workflow, and professional practice. By making transparent the rationale for the narrative and exploratory approach, this review offers a foundational synthesis that can guide future investigations in a rapidly evolving domain.

Future Directions:

1. Empirical validation studies: Pilot studies and controlled trials to evaluate the impact of XR and VLM-assisted workflows on diagnostic accuracy, efficiency, and cognitive load.
2. Usability and workflow integration: Investigations into how immersive interfaces and AI copilots can be seamlessly incorporated into routine cytopathology practice without disrupting existing processes.
3. Training and educational programs: Development of structured XR-based training modules and AI-assisted tutoring systems to support skill acquisition and ongoing professional development.
4. Multi-center and cross-disciplinary collaborations: Expanding pilot implementations across institutions and integrating perspectives from computer science, pathology, and human–technology interaction to refine platforms and best practices.
5. Ethical, legal, and professional considerations: Assessing accountability, transparency, interpretability, and the role of the cytopathologist in augmented workflows to ensure safe and responsible adoption.
6. Iterative design and feedback loops: Leveraging real-world user feedback to iteratively refine XR and VLM systems, improving usability, interpretability, and overall effectiveness in cytopathology.

This combined approach acknowledges the exploratory nature of the field while providing actionable guidance for future research, ensuring that early conceptual insights can translate into practical, evidence-based advancements in diagnostic cytopathology.

## 7. Conclusions

Immersive technologies and VLM-based copilots in cytopathology represent an exciting and rapidly evolving frontier. The evidence reviewed in this article, although largely preliminary and exploratory, suggests that these tools have the potential to augment human expertise, improve training, and support complex workflows. Extended reality platforms allow cytopathologists to navigate multi-scale cellular structures, annotate interactively, and rehearse rare or challenging cases in a controlled environment. Similarly, AI copilots powered by vision–language models provide interpretive scaffolding, organize observations into structured reports, and assist with differential diagnosis, reinforcing rather than replacing human judgment.

The current landscape, however, remains highly fragmented. Most studies are proof-of-concept, pilot implementations, or preprints, reflecting the novelty of the field and the scarcity of peer-reviewed empirical data. The inclusion of preprints and gray literature, while introducing potential selection bias, was necessary to capture the cutting-edge developments and emerging applications that would otherwise be invisible in traditional bibliographic databases. This pragmatic approach allowed us to integrate insights from multiple disciplines, identify recurring themes, and frame a coherent conceptual roadmap despite limited formal validation.

Importantly, the integration of immersive and AI-assisted tools into routine cytopathology practice faces significant challenges. Technical variability, workflow constraints, interoperability issues, and regulatory considerations must be carefully addressed. Human oversight remains essential, as the cytopathologist continues to hold ultimate diagnostic authority. These observations reinforce the copilot paradigm, in which technology acts as a supportive, cognitive layer rather than an autonomous decision-maker. Lessons from radiology and other more mature diagnostic fields show that successful adoption requires careful alignment with clinical workflows, interpretability, and professional accountability—principles that are equally relevant for cytopathology.

Looking ahead, future research should focus on empirical validation, workflow integration, and educational evaluation. The refinement of domain-specific VLMs, incorporation of retrieval-augmented mechanisms, and iterative co-design with cytopathologists will be critical to ensure reliability, usability, and trust. Ethical, regulatory, and organizational frameworks must evolve in parallel, safeguarding patient safety and professional responsibility.

In conclusion, immersive technologies and AI copilots offer a promising, human-centered pathway to enhance cytopathology practice. They can reduce cognitive load, improve visualization of complex cellular structures, and support structured reporting, all while preserving the central role of the cytopathologist. While empirical evidence is still limited, this review provides a conceptual synthesis and practical framework that can guide future studies, pilot projects, and educational initiatives, helping translate early-stage innovations into clinically meaningful and responsible applications.

## Figures and Tables

**Table 1:** Selected immersive technologies in healthcare and education. The table summarizes key platforms, their main features, typical applications, expected benefits, and relevant references. “Key Features” highlights the technological capabilities; “Typical Applications” lists common use cases in clinical practice and education; “Expected Benefits” outlines the advantages or improvements these technologies offer in healthcare and learning contexts.

Technology/Platform	Key Features	Typical Applications	Expected Benefits	References
HoloLens 2 (Microsoft)	AR/MR, holographic overlays, spatial audio, tracking sensors, wireless connectivity	Surgical navigation, anatomy education, digital pathology	Immersive 3D visualization, enhanced spatial understanding, workflow optimization	[39]
Oculus Quest (Meta)	Standalone VR headset, hand tracking, room-scale VR	Surgical simulation, digital pathology, procedural training	Safe skill acquisition, immersive practice, flexible deployment	[40]
Apple Vision Pro (Apple)	AR/MR, high-resolution spatial visualization, gesture and eye tracking	Surgical planning, medical education	High-fidelity immersive experience, spatial computing, educational engagement	[41]
Valve Index (Valve)	High-resolution VR, finger tracking, room-scale, adjustable audio	Training simulations, 3D anatomy	Interactive learning, precise hand tracking, multi-user support	[42]
Google Cardboard (Google)	Lightweight, smartphone-powered VR	Introductory VR training	Low-cost, accessible, scalable for education	[43]
Samsung GearVR (Samsung)	Lightweight, smartphone-powered VR	Entry-level VR training	Affordable, easy-to-deploy, initial VR experience	[44]

**Table 2:** Representative Vision–Language Model (VLM) copilots in biomedical applications. The table summarizes key platforms, their application domains, main features, expected benefits, and relevant references. “Application Domain” indicates the clinical or biomedical context in which the VLM is applied; “Key Features” highlights the technological or functional capabilities; “Expected Benefits” describes the advantages provided to clinicians or researchers, such as support in decision-making or protocol adherence.

Platform	Application Domain	Key Features	Expected Benefits	References
Med-PaLM 2 (Google DeepMind)	Clinical decision support, guideline interpretation	Context-aware reasoning, evidence-based recommendations	Supports decision-making, reduces cognitive load, enhances protocol adherence	[45]
Claude (Anthropic)	Real-time biomedical research, clinical assistance	Retrieval-augmented generation (RAG), context-sensitive prompting	Synthesizes large datasets, supports evidence-informed decisions, enhances research efficiency	[46]
ChatGPT (OpenAI)	Literature summarization, experimental planning, patient information extraction	Fine-tuned NLP for biomedical domains	Accelerates literature review, aids experimental design, extracts patient-relevant information	[47]
BioGPT (Microsoft Research)	Scientific publication mining, molecular and clinical relationship extraction	Pretrained on biomedical corpora, NLP reasoning	Hypothesis generation, relationship extraction, efficient knowledge synthesis	[48]

**Table 3:** Key domains, keywords, and scope of inquiry in immersive and AI-assisted cytopathology. The table outlines the main domains or sources considered in the review, the search keywords or queries used to explore each domain, and the purpose or scope of the inquiry. “Domain/Source” indicates the field or dataset analyzed; “Search Keywords/Queries” lists the terms used to identify relevant literature or technologies; “Purpose/Scope” describes the objective of the search or the aspect of cytopathology being investigated.

Domain/Source	Search Keywords/Queries	Purpose/Scope
LLMs and AI copilots in cytopathology	*Query: ((LLM[Title/Abstract]) OR (“large language model”[Title/Abstract]) OR (VLM[Title/Abstract]) OR (“vision–language model”[Title/Abstract]) OR (copilot[Title/Abstract])) AND (cytopathology[Title/Abstract])*	Explore early-stage research on LLM integration as copilots in cytopathology workflows
Immersive technologies in cytopathology	*((VR[Title/Abstract]) OR (“Virtual Reality”[Title/Abstract]) OR (AR[Title/Abstract]) OR (“Augmented Reality”[Title/Abstract]) OR (MR[Title/Abstract]) OR (“Mixed Reality”[Title/Abstract]) OR (Metaverse[Title/Abstract])) AND (cytopathology[Title/Abstract])*	Identify preliminary studies on immersive tech applications (VR, AR, XR) in cytopathology
Cross disciplinary perspectives	*((“Human–Technology Interaction”[Title/Abstract]) OR (“Medical Informatics”[Title/Abstract]) OR (“Education”[Title/Abstract])) AND (“Digital Pathology”[Title/Abstract])*	Capture broad conceptual insights, workflow integration issues, and implications for training and human–AI collaboration
Gray literature and technical reports	*(“White paper” OR “conference abstract” OR “blog”) AND (“cytopathology” OR “digital pathology”)*	Document pilot projects, educational implementations, and workflow experiments

**Table 4:** Sources and scope of inquiry in immersive and AI-assisted cytopathology. The table summarizes the types of sources reviewed, examples or representative platforms, and their purpose in the analysis. “Type/Source” indicates the category of literature or material analyzed; “Examples/Platforms” provides representative journals, conferences, or tools; “Purpose in Analysis” describes the objective of including these sources in the review, such as consolidating evidence on technology, workflow, or applications in cytopathology.

Type/Source	Examples/Platforms	Purpose in Analysis
Peer-reviewed papers	Scientific journals	Consolidated evidence on technology and workflow
Preprints	arXiv, medRxiv	Emerging trends, prototypes, early implementations
Gray literature	Technical reports, white papers, blogs	Practical implementations, case studies, non-peer-reviewed guidelines
Cross-disciplinary	Computer science, digital pathology, education, HCI	Complementary perspectives and professional implications

**Table 5:** Technologies, contexts, purposes, and boundaries of reviewed studies. The table summarizes selected studies, the technologies and settings used, the purpose or focus of each study, and key observations or limitations. “Study/Authors” identifies the cited work; “Technology/Setting” describes the immersive or AI-assisted tool and its context of use; “Purpose/Focus” outlines the main objective of the study; “Observations/Boundaries” highlights notable outcomes, limitations, or areas requiring further validation.

Study/Authors	Technology/Setting	Purpose/Focus	Observations/Boundaries
Clay et al., 2024 [52]	Immersive VR	VR training for non-specialized medical procedures for caregivers and students	Positive engagement and learning outcomes; highlights need for further empirical validation.
Chance, 2025 [53]	Immersive VR + AI	Support interdisciplinary learning and patient safety in healthcare education	Improved teamwork and adherence to safety principles; evidence is limited.
Lim & Yap, 2024 [54]	Haptic VR	VR simulation for surgical procedure training	Enhances procedural rehearsal and manual dexterity; does not replace real patient experience.
Iqbal et al., 2024 [55]	Immersive VR/AR	Immersive technologies for patient care and training	Improves patient engagement and procedural understanding; complements traditional education.
Hang et al., 2023 [56]	Digital Pathology	Slide scanning and cytopreparations for digital urine cytology	High-resolution imaging improves diagnostic accuracy; fidelity is critical for learning and workflow.
Schwen et al., 2023 [57]	Digital Pathology Labs	Digitization of pathology labs	Enhances workflow standardization and reproducibility; technical and logistical constraints exist.
Osamura et al., 2021 [58]	Computational Cytology	Molecular cytology testing with digital tools	Safe practice and skill acquisition in controlled environments; supports training.
Venkatesan et al., 2021 [59]	Immersive VR/AR	Biomedical applications	Exploration of 3D cellular structures enhances spatial understanding; dependent on system fidelity.
Chen et al., 2018 [60]	AR Microscope + AI	Microscope 2.0 with real-time AI integration	AR overlays and AI assist interpretation; human oversight remains essential.

**Table 6:** Technologies, contexts, purposes, and boundaries of reviewed studies involving large language models (LLMs). The table summarizes selected studies, the LLM technologies and their clinical or educational settings, the main purpose or focus of each study, and key observations or limitations. “Study” identifies the cited work; “Technology/Setting” describes the LLM used and its context; “Purpose/Focus” outlines the primary objective of the study; “Observations/Boundaries” highlights notable outcomes, limitations, or considerations for safe and effective use.

Study	Technology/Setting	Purpose/Focus	Observations/Boundaries
[61]	LLMs	Clinical decision support	Enhances guideline synthesis, organizes clinical information, and supports complex reasoning. Not a diagnostic authority; performance depends on structured and complete inputs.
[62]	LLMs (guideline-aware)	Oncology decision support (NCCN)	Reinforces adherence to standardized care pathways; aids case reasoning in oncology. Requires curated inputs; not validated for independent clinical diagnosis.
[63]	GPT-4	Complex case reasoning	Generates differential diagnoses, summarizes longitudinal patient records, and assists complex reasoning. Susceptible to hallucination and contextual misinterpretation; limited causal grounding; unsuitable for unsupervised diagnosis.
[64,68]	Multimodal VLM/foundation model	Digital pathology assistant	Integrates visual and textual reasoning; supports annotation, exploration, and reporting. Visual encoder quality is critical; LLM layer assists interpretation, not primary inference.
[65]	Whole-slide multimodal foundation model	Pathology workflow integration	Provides interactive guidance, explanatory outputs, and structured reporting support. Dependent on domain-specific pretraining; not a standalone diagnostic system.
[66]	GNNFormer	Cytopathology report generation	Graph-based modeling of cellular morphology and spatial organization; enhances interpretability. Requires domain-aligned inductive biases; general-purpose LLMs insufficient alone.
[67]	Multimodal chatbot AI	Pathology copilot	Supports interactive exploration, report drafting, and user queries. Sensitive to rare entities and prompt formulation; not a primary diagnostic engine.
[69]	PathOrchestra foundation model	Large-scale histopathology tasks	Pretrained on diverse WSIs; performs pan-cancer classification, lesion detection, biomarker assessment. Requires heterogeneous datasets; generalization limited by institutional variability.
[70]	Prov-GigaPath foundation model	Whole-slide digital pathology	Captures cross-institutional variability; supports robust visual feature extraction. Dependent on provenance-aware datasets; not designed for standalone diagnostic decisions.
[71]	PathologyVLM (large vision–language model)	Pathology image understanding	Trained on high-resolution whole-slide images with paired textual annotations; enables cross-scale feature extraction, case retrieval, interactive exploration, and structured reporting. Functions as a workflow-integrated copilot, enhancing interpretability and report standardization while diagnostic authority remains with foundation models and clinicians.
[72]	LLMs (DRAGON benchmark)	Clinical text extraction	Annotates, structures, and normalizes clinical text; assists reporting. Provides interpretive support; human oversight is mandatory.
[73]	RAG-optimized LLMs	Digital cytology support	Delivers up-to-date domain-specific knowledge at inference; enhances training and QA. Reference-driven support only; does not perform independent diagnostic inference.
[74,75,76,77]	Domain-specific multimodal LLM	Histopathology and clinical image interpretation	Proof-of-concept for integrating LLM with visual foundation models; supports report generation and contextual explanation. Not clinically approved; designed for workflow integration; diagnostic authority remains with foundation models and clinicians.

**Table 7:** Comparative overview of cytopathologist tasks, diagnostic challenges, and the potential contribution of extended reality (XR) and vision–language model (VLM)–based copilots in cervical and lung cytology. The table summarizes key features of each cytology type, including specimen type and staining, diagnostic tasks, and challenges. It also outlines how XR and VLM-based tools could support cytopathologists in these domains. “Feature” lists the aspect under consideration; “Cervical Cytology (Pap Smear)” and “Lung Cytology (Sputum/Bronchial Wash)” describe the characteristics, challenges, and potential XR/VLM support for each cytology type.

Feature	Cervical Cytology (Pap Smear)	Lung Cytology (Sputum/Bronchial Wash)
Specimen Type and Staining	Cervical cells stained with Papanicolaou (Pap) technique.	Lung cells from sputum or bronchial wash; typically Pap or Diff-Quik stain.
Role of Cytopathologist	Examine nuclear size, shape, and N/C ratio; Identify dysplastic or malignant clusters; Distinguish atypical from reactive or artifact cells; Issue standardized cytology report (e.g., Bethesda system); Communicate findings to clinical team.	Assess nuclear morphology, cytoplasmic features, and nucleoli; Identify malignant cells amid heterogeneous background; Integrate morphological findings with clinical context; Distinguish malignant cells from inflammatory or reactive cells; Provide structured diagnostic report.
Diagnostic Challenges	Subtle early cellular changes; Small clusters or isolated atypical cells; Artifacts and background debris can mimic dysplasia; Requires high concentration and expertise.	Sparse malignant cells amid mucus, inflammatory cells, and debris; Heterogeneous cell population; Subtle atypia can mimic reactive/inflammatory processes; Requires careful scanning and integrative interpretation.
Extended Reality (XR) Potential	Immersive multi-scale slide exploration; Interactive annotation of suspicious areas; Repeated practice in controlled environment; Collaborative review of rare or ambiguous cases; Reduces cognitive load and enhances spatial understanding [52,53,54,55,59,79,80,91,92].	Immersive slide navigation and multi-scale visualization; Highlighting clusters in heterogeneous backgrounds; Repeated examination of sparse malignant cells; Facilitates collaborative case review; Reduces interpretive errors in complex specimens [52,53,54,55,59,79,80,91,92].
Vision–Language Model (VLM) Potential	Automatic extraction of structured information from images; Drafting preliminary reports or summaries; Guideline-aligned documentation (e.g., Bethesda categories); Assisting differential diagnosis and highlighting features of concern [64,65,66,67,68,69,70,71,78,95,96].	Extract and summarize cellular features and contextual information; Aid report drafting and differential diagnosis; Prioritize suspicious regions for human review; Provide interactive explanations for challenging backgrounds [64,65,66,67,68,69,70,71,78,95,96].
Synergistic Effect of XR + VLM	XR guides visual exploration and highlights features; VLM organizes findings into structured reports; Supports training, education, and workflow without replacing human judgment; Enhances interpretability and professional accountability [61,62,63,85].	XR enhances detection of isolated malignant cells; VLM structures information and assists in report generation; Supports cognitive load reduction and workflow efficiency; Maintains cytopathologist as ultimate decision-maker [61,62,63,85].

## Data Availability

No new data were created or analyzed in this study. Data sharing is not applicable to this article.

## Supplementary Materials

The following supporting information can be downloaded at: https://www.mdpi.com/article/10.3390/jimaging12030100/s1, Section S1: Further details of the study selection process; Section S2: Consensus and Supplementary Details on Included Sources
