# Supplementary material for: The Augmented Cytopathologist: A Conceptual Exploratory Narrative Review on Immersive and Vision–Language Models Tools in Digital Pathology
Source: J Imaging. 2026 Feb 26;12(3):100. doi: 10.3390/jimaging12030100 (PMC13027800; doi:10.3390/jimaging12030100)
Supplement: Supplementary file 1 [file jimaging-12-00100-s001.zip › jimaging-4017949-supplementary.pdf]

Review

SUPPLEMENTARY MATERIAL

The Augmented Cytopathologist: A Conceptual Exploratory Narrative Review on Immersive and Vision–Language Models tools in Digital Pathology

Enrico Giarnieri [1], Andrea Lastrucci [2], Alberto Ricci [3], Pierdonato Bruno [3] and Daniele Giansanti [4,*]

[1] Facoltà di Medicina e Psicologia, Sede Ospedale S. Andrea via di Grottarossa 1035, Università Sapienza, 00189 Roma, Italy; enrico.giarnieri@uniroma1.it
[2] Dipartimento Professioni Tecnico Sanitarie e della Riabilitazione, Azienda Ospedaliero-Universitaria Careggi, 50134 Florence, Italy; andrea.lastrucci@unifi.it
[3] Respiratory Disease Unit, Sant'Andrea University Hospital, Sapienza University of Rome, Via di Grottarossa 1035, 00189 Rome, Italy; alberto.ricci@uniroma1.it (A.R.); pierdo.bruno@gmail.com (P.B.)
[4] Centro Nazionale IAHTA, Istituto Superiore di Sanità, via Regina Elena 299, 00161 Rome, Italy
* Correspondence: daniele.giansanti@iss.it

S.1 Further details of the study selection process

S.1.1 The consensus report

In the results section, the findings of the review are structured around the sources retained for thematic synthesis. For transparency, a Consensus Report (CR) was compiled for each study or source type, capturing the rationale for its inclusion and the conceptual domains it informs. Table S1 summarizes these entries, indicating the source type (per Table 4), the domains addressed, and a verbal justification.

This approach allows readers to trace how each source contributed to understanding emerging immersive technologies and AI copilots in cytopathology, including pilot projects, early implementations, and cross-disciplinary insights. Even non-peer-reviewed sources, such as preprints, technical reports, and platform documentation, were assessed for their relevance to key domains—technological capabilities, workflow and educational applications, professional implications—without implying formal methodological quality grading. The preparation of the CR followed a concept-driven and iterative approach, consistent with the exploratory nature of a CENR. Each source was independently assessed by two reviewers (D.G. and E.G.) for its ability to inform the conceptual framework. Any discrepancies were resolved through discussion, ensuring a shared interpretive consensus.

Notably, even sources outside traditional peer-reviewed literature were included and justified, emphasizing conceptual relevance rather than methodological quality. This approach allowed the review to capture early pilot implementations, experimental platforms, and cross-disciplinary perspectives that are critical to understanding the evolving role of immersive technologies and AI copilots in cytopathology workflows. By providing a verbal justification for each entry, the CR serves as a transparent bridge between the evidence gathered and the thematic synthesis presented in the results, highlighting how each source contributes to the overall interpretive framework.

This table summarizes all sources included in the thematic synthesis, indicating the source type (as defined in Table 4), the domains addressed, and a verbal justification for

Academic Editors: Leonardo Rundo and William E. Higgins

Received: 15 November 2025
Revised: 16 February 2026
Accepted: 18 February 2026
Published: date

Copyright: © 2026 by the authors. Submitted for possible open access publication under the terms and conditions of the Creative Commons Attribution (CC BY) license.

inclusion. Each entry was assessed through a concept-driven and iterative Consensus Evaluation Narrative Report (CENR), ensuring transparency and traceability. Sources were evaluated for their ability to inform the conceptual framework of immersive technologies and AI copilots in cytopathology, including technological capabilities, workflow and educational applications, and professional implications. Both peer-reviewed and non-peer-reviewed sources (preprints, technical reports, and platform documentation) were included based on conceptual relevance rather than formal methodological quality. Each source was independently reviewed by two evaluators (D.G. and E.G.), with discrepancies resolved through discussion to achieve interpretive consensus. By providing a verbal justification for each entry, this table serves as a transparent bridge between collected evidence and the thematic synthesis presented in the results, highlighting how each source contributes to the overall interpretive framework.

Table S1. Consensus report of the selected studies.

#	Citation	Type (per Table 4)	Domain(s) addressed	Verbal justification
52	Clay, C.J., Budde, J.R., Hoang, A.Q., et al. (2024). Front. Virtual Real. 5:1402093	Peer-reviewed paper	Technological capabilities; Workflow/Educational applications	Reviews VR training effectiveness for non-specialized medical procedures. Relevant conceptually for understanding immersive educational interventions applicable to cytopathology training and workflow integration.
53	Chance, E.A. (2025). BMC Med Educ 25, 1039	Peer-reviewed paper	Workflow/Educational applications; Professional implications	Narrative review on AI + VR impact on interdisciplinary learning and patient safety. Provides insights on professional roles, collaborative learning, and early adoption of immersive and AI-assisted tools in healthcare education.
54	Lim Zheng Jie & Kian Meng Yap (2024). arXiv:2411.05148	Preprint	Technological capabilities; Workflow/Educational applications	Presents haptic VR simulation for surgery training. While early-stage and non-peer-reviewed, it illustrates emerging technological solutions for skill acquisition and immersive learning, conceptually relevant to cytopathology education.
55	Iqbal, A.I., Aamir, A., Hammad, A., et al. (2024). J Prim Care Community Health 15	Peer-reviewed paper	Technological capabilities; Workflow/Educational applications	Explores VR and AR applications in patient care and training. Adds understanding of the readiness of immersive tools for clinical and educational integration, relevant for early-stage workflow adaptations.

56	Hang JF, Ou YC, Yang WL, et al. (2023). J Pathol Inform 15:100346	Peer-reviewed paper	Technological capabilities; Professional implications	Comparative evaluation of slide scanners and cytopreparations. Highlights practical constraints and technological maturity, informing professional implications for adopting digital and AI-assisted cytopathology workflows.
57	Schwen, L.O., Kiehl, T.-R., Carvalho, R., et al. (2023). arXiv:2306.03619	Preprint	Technological capabilities; Professional implications	Reviews lessons from digitization of pathology labs. Conceptually valuable for understanding operational barriers, workflow readiness, and potential for AI/immersive tool adoption.
58	Osamura RY, Matsui N, Kawashima M, et al. (2021). Acta Cytol. 65(4):342–347	Peer-reviewed paper	Technological capabilities; Professional implications	Discusses computational technologies for molecular cytology testing. Provides insights into technological feasibility and professional integration challenges, informing conceptual framework for AI copilot adoption.
59	Venkatesan M., Mohan H., Ryan J.R., et al. (2022). Cell Rep Med 2(7):100348	Peer-reviewed paper	Technological capabilities; Workflow/Educational applications	Explores VR and AR applications in biomedical contexts. Conceptually relevant for mapping emerging immersive technologies to cytopathology training and workflow optimization.
60	Chen P.-H.C., Gadepalli K., MacDonald R., et al. (2018). arXiv:1812.00825	Preprint	Technological capabilities; Workflow/Educational applications	Introduces AR microscope with real-time AI integration. Highlights early-stage experimental platform that exemplifies integration of immersive and AI tools, conceptually important for cytopathology workflow innovation.
61	Jia Li, Z., Zhou, Z., Lyu, H., Wang, Z. (2025). Intelligent Medicine, 5(1), 1-4	Peer-reviewed paper	Technological capabilities; Professional implications	Discusses LLM-powered clinical decision support for enhancing or substituting human expertise. Conceptually relevant for evaluating AI copilots' role in augmenting clinical reasoning, aligning with emerging AI-assisted workflows in cytopathology.
62	Kaiser KN, Hughes AJ, Yang AD, et al. (2025). Surgery 182:109267	Peer-reviewed paper	Technological capabilities;	Explores LLMs as clinical decision support for pancreatic

			Professional implications	adenocarcinoma. Provides insights into practical deployment of LLMs, professional trust, and workflow integration—informative for conceptual mapping of AI copilots in pathology.
63	Eriksen A.V., Moller S., Ryg J. (2024). NEJM AI, 1(1)	Peer-reviewed paper	Technological capabilities; Professional implications	Evaluates GPT-4 in diagnosing complex clinical cases. Conceptually illustrates limitations, performance boundaries, and professional reliance on AI, informing safe and responsible adoption of LLMs in cytopathology.
64	Lu M.Y., Chen B., Williamson D.F.K., et al. (2023). arXiv:2312.07814	Preprint	Technological capabilities; Workflow/Educational applications	Introduces a foundational multimodal vision-language AI assistant for pathology. Faces the integration of visual slide data and natural language interaction, supporting diagnostic reasoning, reporting assistance, and educational augmentation. Conceptually central for framing AI copilots in cytopathology workflows.
65	Ming Y. Lu, Bowen Chen, Williamson D.F.K., et al. (2023). arXiv:2312.07814	Preprint	Technological capabilities; Workflow/Educational applications	Introduces a multimodal vision-language AI assistant for pathology. Provides early-stage evidence of immersive AI integration for educational and workflow augmentation, useful for conceptual synthesis of AI copilots in cytopathology.
66	Tong Ding, Wagner S.J., Song A.H., et al. (2024). arXiv:2411.19666	Preprint	Technological capabilities; Workflow/Educational applications	Describes a multimodal whole-slide foundation model. Conceptually important for exploring experimental platforms that integrate visual and textual data, relevant to training, diagnostic assistance, and workflow support.
67	Yang-Fan Zhou, Kai-Lang Yao, Wu-Jun Li (2023). arXiv:2303.09956	Preprint	Technological capabilities; Workflow/Educational applications	Graph-based framework for cytopathology report generation. Provides an early conceptual example of AI-assisted workflow optimization, showing potential impact on report accuracy and training processes.

68	Lu M.Y., Chen B., Williamson D.F.K., et al. (2024). Nat Med 30, 863–874	Peer-reviewed paper	Technological capabilities; Workflow/Educational applications; Professional implications	Presents a visual-language foundation model for computational pathology. Combines peer-reviewed rigor with practical insights into AI-assisted diagnostics, bridging technological capability, workflow integration, and professional implications.
69	Yan, F., Wu, J., Li, J., et al. (2025). PathOrchestra: a comprehensive foundation model for computational pathology with over 100 diverse clinical-grade tasks. npj Digit. Med. 8, 695.	Peer-reviewed paper	Technological capabilities; Workflow/Educational applications; Professional implications	Describes a large-scale foundation model for pathology covering diverse tasks. Conceptually relevant for understanding integration of multimodal AI into diagnostic workflows, training, and professional practice.
70	Xu, H., Usuyama, N., Bagga, J., et al. (2024). A whole-slide foundation model for digital pathology from real-world data. Nature 630, 181–188.	Peer-reviewed paper	Technological capabilities; Workflow/Educational applications	Introduces a whole-slide model trained on real-world data. Highlights practical implementation, generalization, and workflow implications for cytopathology, supporting conceptual synthesis on AI copilots.
71	Dai, D., Zhang, Y., Yang, Q., et al. (2025). PathologyVLM: a large vision-language model for pathology image understanding. Artif Intell Rev 58, 186.	Peer-reviewed paper	Technological capabilities; Workflow/Educational applications	Demonstrates VLM for pathology image understanding. Provides insight into AI-assisted diagnostic support, relevant for conceptualizing AI copilots' augmentation of cytopathologist tasks.
72	Bosma, J.S., Dercksen, K., Builtjes, L., et al. (2025). The DRAGON benchmark for clinical NLP. npj Digit. Med. 8, 289.	Peer-reviewed paper	Technological capabilities	Benchmark for clinical NLP tools. Conceptually supports evaluation of AI models' performance, informing considerations on AI reliability and role in workflow support.
73	Al-Asi, H., Reynolds, J.P., Agarwal, S., et al. (2025). Enhancing Thyroid Cytology Diagnosis with RAG-Optimized LLMs and Pathology Foundation Models. arXiv:2505.08590	Preprint	Technological capabilities; Workflow/Educational applications	Explores LLMs optimized for cytology tasks. Provides early-stage experimental insight into AI copilots' potential to augment diagnostic accuracy and workflow in cytopathology.
74	Sellergren A., Kazemzadeh S., Jaroensri T., et al. (2025). MedGemma Technical Report. arXiv:2507.05201	Preprint	Technological capabilities; Workflow/Educational applications	Technical report on MedGemma platform. Conceptually included to capture early-stage implementations of AI copilots in medical workflows.

75	Google Health. MedGemma Developer Foundations. URL: https://developers.google.com/health-ai-developer-foundations/medgemma	Grey literature / Online platform	Technological capabilities; Workflow/Educational applications	Online documentation of MedGemma. Provides practical details on platform features, model architecture, and deployment, useful for understanding AI copilots. *Implicitly suggested by Reviewer 5*
76	Google Health. MedGemma on HuggingFace. URL: https://huggingface.co/google/medgemma-1.5-4b-it	Grey literature / Online platform	Technological capabilities; Workflow/Educational applications	Platform hosting and model details for MedGemma. Conceptually relevant for exploring model accessibility, experimentation, and integration in educational or diagnostic workflows. *Implicitly suggested by Reviewer 5*
77	Google Health. MedGemma Model Card v1. URL: https://developers.google.com/health-ai-developer-foundations/medgemma/model-card-v1	Grey literature / Online platform	Technological capabilities; Workflow/Educational applications	Model card provides transparency, limitations, and intended use. Supports conceptual understanding of safe and responsible AI deployment, aligned with integrative CENR goals. *Implicitly suggested by Reviewer 5*

S.1.2 Study Selection Process (PRISMA-inspired overview)

Although this review did not follow a formal systematic review methodology and the PRISMA checklist was not applied, a structured, consensus-driven approach was used to identify and select relevant sources. The process ensured transparency and traceability while remaining aligned with the exploratory and integrative nature of the CENR. A PRISMA-inspired diagram (Figure S1) is provided to visually summarize the selection flow.

Initial identification: 132 candidate sources were retrieved from bibliographic databases (PubMed, Scopus, Web of Science), preprint servers (arXiv, medRxiv), grey literature, and cross-disciplinary platforms.

After removal of duplicates: 108 unique records remained.

For peer-reviewed articles, an initial pre-screening was conducted based on titles and abstracts. For web-based resources and project websites, relevance was assessed through a content-based pre-screening, considering page headings, introductory descriptions, and contextual information.

During the initial pre-screening phase, 59 records (including both peer-reviewed articles and web-based resources) were excluded because they were not relevant to the key conceptual domains of this review, namely technological capabilities, workflow and educational applications, professional implications, and the role of AI-based copilots in cytopathology.

Full-text/content assessment: 23 sources were excluded because they did not provide sufficient conceptual information or were outside the scope of cytopathology.

Final inclusion: 26 sources (citations 52–77) were included in the thematic synthesis and are reported in the Consensus Report (Table S1).

All selections were made by consensus, with each source evaluated for its conceptual relevance rather than formal methodological quality. This approach ensured that the

CENR captured the diversity of emerging discussions, pilot implementations, and experimental platforms in immersive technologies and AI copilots for cytopathology.

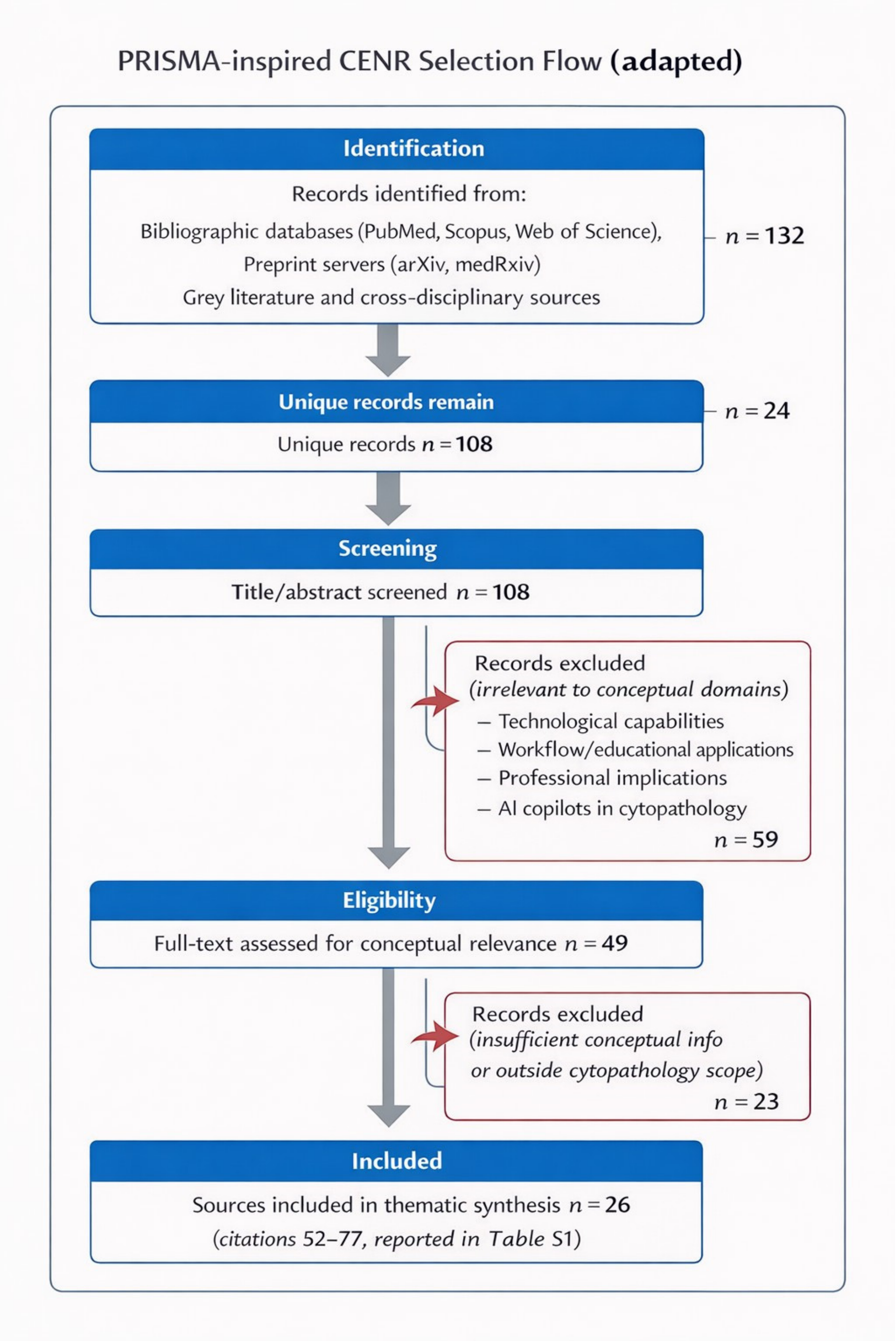

Figure S1. PRISMA-inspired selection flow for the Consensus Evaluation Narrative Report (CENR). This flow diagram summarizes the structured, consensus-driven process used to identify, screen, and select sources included in this exploratory narrative review. Although a formal systematic review methodology was not applied and the PRISMA checklist was not followed, the diagram is inspired by the PRISMA 2020 framework and adapted to reflect the heterogeneous nature of the included sources. Peer-reviewed articles were screened based on titles/abstracts and full-text content, while web-based resources and project websites underwent a content-based screening using headings, introductory descriptions, and contextual information. Selection and eligibility were guided by conceptual relevance to technological capabilities, workflow and educational applications, professional implications, and the role of AI-based copilots in cytopathology. All decisions

were made by consensus to ensure transparency and traceability while remaining aligned with the integrative scope of the CENR.

S.2 Consensus and Supplementary Details on Included Sources

Notably, a Consensus Report (CR) was also prepared for each source included in the other sections, particularly for websites and grey literature, which are reported below.

Table S2. Consensus report for grey literature sources included outside the Results section. This table summarizes non–peer-reviewed sources, primarily web-based resources and grey literature, evaluated through a Consensus Evaluation Narrative Report (CENR). For each source, the table reports the material type (as defined in Table 4) and a verbal justification describing its conceptual, educational, technological, or contextual contribution to the review. Inclusion was based on relevance to immersive technologies and vision–language model–based copilots in cytopathology, authoritative origin, and transparency, in line with the aims of this conceptual exploratory narrative review.

#	Type (per Table 4)	Verbal justification
8	Web resource	Provides a comprehensive virtual microscopy platform, allowing users to explore digital slides and imaging techniques. This resource supports the conceptual understanding of digital cytopathology workflows and immersive visualization, forming a foundational reference for the CENR.
9	Web resource	Offers structured histology guides that explain tissue morphology and staining methods. These materials inform background knowledge essential for interpreting cytopathological images and contextualize educational and workflow-related discussions in the review.
10	Web resource	Features virtual microscopy tools with case examples, enabling an interactive exploration of specimen analysis. This resource enriches the conceptual framework by illustrating practical applications of immersive learning environments and digital pathology techniques.
39	Web resource	Official Microsoft HoloLens 2 site. Provides authoritative technical specifications, developer guidance, and real-world applications. Essential for understanding the capabilities of cutting-edge AR hardware and its potential to enhance immersive cytopathology training and workflow simulations.
40	Web resource	Oculus Quest 2 official site. Presents detailed hardware features, software ecosystem, and VR use cases. Crucial for contextualizing immersive VR solutions in healthcare education and evaluating their applicability to interdisciplinary training scenarios.
41	Web resource	Apple Vision Pro official site. Offers insights into next-generation mixed-reality technology, highlighting platform capabilities, ergonomics, and potential for integration into clinical and educational immersive environments.

42	Web resource	Valve Software official page. Provides comprehensive information on VR hardware, developer tools, and ecosystem support. Relevant for understanding high-performance VR platforms that could underpin experimental cytopathology simulations or collaborative training modules.
43	Web resource	Google Cardboard official page. Showcases low-cost, accessible VR hardware, emphasizing scalability and broad educational reach. Important for contextualizing the feasibility of immersive interventions in resource-constrained or widely distributed training settings.
44	Web resource	Official Samsung site for the Galaxy VR headset. Provides technical specifications, user guidance, and real-world applications. Relevant for understanding commercially available immersive hardware and its potential role in enhancing experiential learning and remote cytopathology training.
45	Web resource	Google Research Med-PaLM project page. Presents information on the development of medical large language models, research aims, and early applications. Crucial for contextualizing AI copilots in clinical decision support and exploring their conceptual integration into cytopathology workflows.
46	Web resource	Anthropic Claude official site. Offers insight into a state-of-the-art AI assistant, including capabilities, intended use cases, and safety considerations. Relevant for understanding the conceptual potential of AI copilots in augmenting professional expertise and supporting decision-making in cytopathology.
48	Web resource	Microsoft Research BioGPT page. Although not a peer-reviewed study, it illustrates the development and capabilities of a large language model specifically designed for biomedical text generation and mining. Its inclusion provides background on AI copilots' potential to support information synthesis, clinical decision support, and knowledge augmentation in biomedical contexts, relevant to conceptualizing AI-assisted workflows in cytopathology.
49	Web resource	Market analysis reports on immersive technologies and AI in healthcare. Although not peer-reviewed, these sources provide contextual information on adoption trends, investment priorities, and commercial deployment of VR/AR and AI-assisted platforms. Inclusion supports understanding the broader environment in which AI copilots and immersive tools are being integrated, offering background for workflow planning and strategic perspectives in cytopathology.
50	Web resource	Industry websites and news portals tracking AI and biomedical technology markets. These sources offer

		insights into emerging products, competitive landscape, and projected growth, which inform conceptual framing of technology adoption and potential professional impact for cytopathologists.
51	Web resource	MDPI Journal of Imaging instructions page. While not a study, this source provides context on publication standards and scope for imaging research, relevant to framing the methodological and reporting expectations for studies referenced in the CENR. Its inclusion helps situate the review within the broader landscape of imaging-focused scholarly communication.
97	Web resource	PubMed query openly accessible, checked in real-time; provides an updated overview of publications on large language models. Its use is consistent with MDPI policies and with our previous practice in other reviews.
98	Web resource	PubMed query openly accessible, checked in real-time; provides an updated overview of publications on immersive technologies (VR, XR, AR) in healthcare. Its use is consistent with MDPI policies and with our previous practice in other reviews.
